# Preventing Respiratory Viral Diseases with Antimicrobial Peptide Master Regulators in the Lung Airway Habitat

**DOI:** 10.3390/clinpract13010012

**Published:** 2023-01-16

**Authors:** Piyush Baindara, Sriradha Ganguli, Ranadhir Chakraborty, Santi M. Mandal

**Affiliations:** 1 Department of Radiation Oncology, University of Missouri, Columbia, MO 65211, USA; 2 OMICS Laboratory, Department of Biotechnology, University of North Bengal, P.O. NBU, Siliguri 734013, West Bengal, India; 3 Department of Biotechnology, Indian Institute of Technology Kharagpur, Kharagpur 721302, West Bengal, India

**Keywords:** lung peptides, lung airways, antimicrobial peptides, antiviral, gene expression, protein-protein interaction network

## Abstract

The vast surface area of the respiratory system acts as an initial site of contact for microbes and foreign particles. The whole respiratory epithelium is covered with a thin layer of the airway and alveolar secretions. Respiratory secretions contain host defense peptides (HDPs), such as defensins and cathelicidins, which are the best-studied antimicrobial components expressed in the respiratory tract. HDPs have an important role in the human body’s initial line of defense against pathogenic microbes. Epithelial and immunological cells produce HDPs in the surface fluids of the lungs, which act as endogenous antibiotics in the respiratory tract. The production and action of these antimicrobial peptides (AMPs) are critical in the host’s defense against respiratory infections. In this study, we have described all the HDPs secreted in the respiratory tract as well as how their expression is regulated during respiratory disorders. We focused on the transcriptional expression and regulation mechanisms of respiratory tract HDPs. Understanding how HDPs are controlled throughout infections might provide an alternative to relying on the host’s innate immunity to combat respiratory viral infections.

## 1. Introduction

The innate immune system is the human body’s initial line of defense against microorganisms such as bacteria, fungi, and viruses [1]. The innate immune system plays a significant role in protection, especially in the respiratory tract, which is constantly exposed to external substances and pathogenic microorganisms. HDPs are a critical component of the innate immune system and are high in cationic amino acid residues which are involved in both antimicrobial and immunomodulatory activities [2,3]. They are frequently amphipathic and range in size from a few hundred amino acid residues to several thousand [4]. HDPs are also found on the mucosal surfaces of the gastrointestinal, urogenital, and respiratory tracts in animals, and they have a pleiotropic effect on both innate and adaptive immune responses [5,6,7]. These multifunctional peptides can guard against bacteria, viruses, fungi, parasites, and protozoa and also regulate inflammation, wound healing, and adaptive immune response [8,9,10]. Defensins, cathelicidins, lactoferrin, lysozyme, secretary leucoprotease inhibitor (SLPI), and chemokine ligand are HDPs produced in the respiratory tract [11]. Lysozyme, a cell-wall-disintegrating enzyme; lactoferrin, an iron-chelating protein; SLPIs, anti-elastase and defensins; and cathelicidins, membrane permeabilizing peptides, are all secreted in respiratory secretions. Antimicrobial proteins, such as bactericidal permeability-increasing protein (BPI), surfactant proteins A–D, and other collectins are also present [11]. These HDPs play a role in pathogen clearance as well as immune response modulation during respiratory infections in the normal lung (Table 1). Additionally, HDP expression is triggered and elevated during infections to protect the host from pathogens which suggested a tight regulation of HDPs in host protection [12]. Furthermore, HDPs chemotactically attract immune cells and modify cellular processes to combat infectious pathogens by engaging with signaling pathways implicated in inflammation and disease progression [13,14,15]. The current study reviewed and explored the regulation and expression of HDPs in the respiratory tract. Interestingly, COVID-19, an ongoing pandemic disease, is also a respiratory disease that is primarily characterized by pneumonia and severe lung inflammation [16]. In the present study, we proposed that respiratory tract HDPs could have a therapeutic role in battling respiratory infectious diseases, such as COVID-19.

## 2. Major Antimicrobial Peptides Expressed in Lung Airways

### 2.1. Defensins

Human β defensins (HBDs) 1, 2, 3, and 4 have been found to have overlapping expression patterns. HBD1 is produced and expressed in the lung airway epithelia, which are in direct contact with ambient microflora [17]. Microbial compounds, such as lipopolysaccharide (LPS) and peptidoglycan, have been shown to increase HBD1 expression [32]. HBD’s have been detected in various organs other than the lungs, such as the heart, liver, lung tumor tissues, and stomach epithelial cells, even though their expression patterns overlap [33,34]. Furthermore, pathogen-derived compounds, cytokines, and chemokines produced by the immune system or injured cells all influence HBD expression [35]. It has been found that when immune cells are exposed to bacteria, LPS, IFN-γ, and IL-6, the expression of HBDs increases [36,37]. In recent studies, human defensins were reported to have antiviral activities against SARS-CoV-2 [38]. Additionally, HNP-1, retrocyclin, and human intestinal defensin5 were shown to reduce the viral infection by blocking the viral entry and were suggested as valuable therapeutic tools to combat SARS-CoV-2 infection [39,40]. Overall, defensins are reported to have antiviral activity against both enveloped and non-enveloped viruses via direct activity and indirectly via immunomodulatory activities [41].

### 2.2. Cathelicidin

The only cathelicidin generated by a vitamin-D-dependent antibacterial mechanism in humans is LL37 [42,43]. LL37, like defensins, is generated as a pre-propeptide in epithelial cells and is involved in the first immune response to a variety of infections [44]. Proinflammatory cytokines and growth hormones, such as the active form of vitamin D, regulate LL37 expression [45]. LL37 attracts neutrophils, monocytes, dendritic cells, and T cells, and it is quickly secreted by epithelial cells and leukocytes during infection in the airways [44]. LL37 increases the synthesis of IL-6 in human dendritic cells and acts as both an anti- and proinflammatory agent during the early stages of an infection’s immune response [46]. Individuals with cathelicidin-deficient neutrophils have been observed to be more susceptible to viral infections [44]. It recent studies, it has been shown that LL37 has direct activity against human rhinovirus and respiratory syncytial virus, and can protect against respiratory infections caused by these viruses in both mice and humans [47,48]. Interestingly, cathelicidins represent an inducible therapeutic target for fighting against viral infections.

### 2.3. Lactoferrin

Lactoferrin is an iron-binding glycoprotein found in breast milk, tears, vaginal secretions, gut-lining fluids, cervical mucus, saliva, and respiratory secretions. Lactoferrin, like other innate immunity proteins, is a cationic protein with antibacterial and anti-inflammatory effects [49]. Neutrophils secrete lactoferrin at the site of inflammation, which aids in host defense and immunological regulation at mucosal surfaces [50]. Lactoferrin has been shown to have potent antibacterial activity against clinical *E. coli*, *S. aureus*, and mucoid *P. aeruginosa* strains isolated from cystic fibrosis patients’ airways [51]. Next, lactoferrin has been shown to have synergistic antibacterial activities against bacteria when combined with other respiratory tract immune proteins, such as lysozyme and SLPI [52]. Lactoferrin has been shown to exhibit antiviral activity against a variety of viruses, including HIV, cytomegalovirus (CMV), and hepatitis B and C, and to have therapeutic promise when combined with interferon [53,54]. Lactoferrin has also been found to prevent inflammatory cells from infiltrating the lungs during pneumonia when taken orally [55]. Lactoferrin is a major inflammatory protein that has been shown to inhibit LPS-induced IL-8 production and peptidoglycan binding characteristics in human endothelial cells [56]. According to a recent study, lactoferrin consumption directly protects the host by inhibiting viral attachment and replication in the cell while also increasing systemic immune activities [57]. Conclusively, lactoferrin has potential antiviral and immunomodulatory properties to protect against respiratory viral infection and is suggested to be used as a nutraceutical [58]. Interestingly, lactoferrin confirmed to have protective effects and reduced respiratory tract infections in controlled randomized clinical trials via modulation of inflammation and immune response [59,60].

### 2.4. Secretory Leucoprotease Inhibitor (SLPI)

SLPI is a non-glycosylated protein that is expressed on the mucosal surface of epithelial cells in the respiratory system by macrophages, neutrophils, and mucosal epithelial cells. SLPI, as well as lysozyme, lactoferrin, and other innate immunity proteins found in respiratory and nasal secretions, is abundant in BAL [61,62]. The serine protease inhibitor SLPI also protects from neutrophil elastase, which is released by neutrophils during infection and inflammation [63]. SLPI is a multifunctional peptide that acts as an antibacterial, antiviral, and anti-inflammatory peptide [64]. In an immunoglobulin G (IgG) immune complex model of acute lung damage, SLPI is found to decrease neutrophil recruitment and thus inflammation in the lungs [65]. Prior treatment with SLPI effectively reduced inflammation in both the liver and the lungs of a mouse model of hepatic ischemia/reperfusion injury [66]. The inactivation of SLPI has also been linked to an increase in lung parenchymal inflammation, tissue destruction, and pneumonia [67]. The oxidative-stress-sensitive protein SLPI is found in respiratory cell linings, which have mechanisms to alleviate oxidative stress. Nuclear factor (erythroid-derived 2)-like 2 (Nrf2) is an oxidative stress regulator that also regulates SLPI expression. Sulforaphane (SFN), an isothiocyanate found in cruciferous vegetables, has recently been shown to boost Nrf2 activity, and consequently, supplementation with SFNs results in increased SLPI secretion in the nasal mucosa [68].

### 2.5. Lysozyme

Lysozyme is a basic antimicrobial protein that kills bacteria by disrupting the glycosidic connection between N-acetylglucosamine and N-acetylmuramic acid residues in peptidoglycan, which is a component of the bacterial cell wall. Both phagocytic and secretary neutrophils retain lysozyme, which is also generated by monocytes, macrophages, and the respiratory epithelium [69]. In the respiratory tracts of lysozyme-expressing transgenic mice, increased bacterial killing is observed. Furthermore, increased lysozyme concentration is found to link to lower systemic bacterial infection and increased in vivo survival [70]. It was demonstrated in a different in vivo study employing tracheal xenograft airways with or without submucosal glands that lysozyme secretary glands efficiently clear the bacterial load. Additionally, Immuno-depletion experiments revealed a strong antibacterial role for lysozyme in lung airways [71]. In a recent study, lysozyme was reported to have protective effects against SARS-CoV-2 in human corneal epithelial cells [72]. Interestingly, niclosamide-lysozyme particles were revealed to have potential anti-coronavirus activities and were suggested to develop as a therapeutic agent [73]. Overall, due to its potential antiviral activities and immunomodulatory properties, lysozyme is suggested as a promising therapeutic agent [74].

### 2.6. Lactoperoxidase

Lactoperoxidase (LPO) is a mammalian heme peroxidase that is released from the mucosal membrane of the airways and has been shown to reduce respiratory infections [30]. The presence of the LPO system and its role in the host defense has been investigated in human airways and tissue secretions [75]. LPO causes the oxidation of iodine, which is reported to increase the antiviral defense of respiratory mucosal surfaces [76]. Next, LPO-produced hypothiocyanite and hypoiodite were shown to have anti-influenza activity and suggested the development of an LPO-based antiviral system to protect against airway infections [77]. LPO is reported to combat the influenza virus in mice upon oral administration by reducing the infiltration of inflammatory cells in the lung [55]. Overall, LPO-based antiviral systems could be developed as an efficient alternative to combat respiratory viral infections to protect the lung airways.

### 2.7. CCL20

Chemokine ligand 20 (CCL20) has an antiparallel sheet core structure, charge distribution, and adaptive immunological signaling via a highly selective CCR6 receptor, all of which have structural and functional parallels with HBDs. CCL20 has been found to have dual functions in innate and adaptive immunity and is regulated by inflammatory mediators expressed in the airway epithelium [31]. Neutrophils have been shown to create CCL20, which is controlled by inflammatory cytokines, such as IL-1 and TNF-, via the NFB pathway [78,79]. CCL20 levels were shown to be higher in cystic fibrosis patients’ BAL compared to healthy BAL, implying that CCL20 plays a function in the respiratory immune defense [31]. CCL20 was reported as having anti-HIV-1 activity in the human female reproductive system via direct interaction with the virus [80]. Furthermore, increased levels of CCL20 are found in COVID-19 patients when compared to healthy counterparts [81]. This suggested a possible virally induced expression regulation of CCL20, which could be therapeutically targeted to protect lung airways against respiratory viral infections.

## 3. Regulation of AMPs Expression in the Respiratory Tract and Innate Immunity

Defensins and cathelicidins are the principal AMPs in the respiratory tract, and they are produced by a variety of cell types, including respiratory epithelial cells, neutrophils, and alveolar macrophages [82]. The main source of α-defensins is neutrophil recruitment in the lungs during infection or inflammation. The local expression of α-defensin (HD-5) is also influenced by respiratory epithelium and submucosal gland cells [83]. Other than α-defensins, non-goblet and serous cells primarily express β-defensins in epithelial cells [84]. Respiratory epithelial cells, alveolar macrophages, monocytes, and dendritic cells all express HBD1 constantly. The expression of hBD3, hBD4, hBD6, and hBD9 genes in respiratory epithelial cells is also modest under baseline circumstances [84,85,86]. Defensin expression has been shown to increase significantly under infection and inflammatory situations [87,88,89].

The expression of human cationic AMPs is both constitutive and inducible, which include HBD2, 3, 4, and LL37 [90,91,92]. LPS, lipoteichoic acid (LTA), TNF, IL-1β, and INF-γ are among the most studied inflammatory mediators that regulate the expression of HDPs [93,94]. Although the processes of AMP induction are poorly known, the HBD2 gene has been demonstrated to be induced by LPS in human airway epithelial cells [95]. In addition to LPS, cationic AMPs have been found to bind LTA, which results in the generation of TNF-α and IL-6 [96]. HBD1, 2, 3, and 4 genes were also found to be regulated by retinoic acid (RA) [97]. Next, the expression induction of HBD2, 3, and 4 is mediated by high Ca^2+^ concentration, proinflammatory cytokines, phorbol myristate acetate (PMA), and bacteria [97]. Furthermore, activation of the NFkβ, AP-1, JAK2, and STAT3 signaling pathways regulates the expression of defensins and cathelicidin genes [35]. In paneth cells, transcription factor-4 (TCF-4) and Wnt signaling pathway transcription factor govern the transcription of human defensins HD5 and HD6 genes [98]. Moreover, HBD3, which is produced in the respiratory airways during infection, is claimed to cause transactivation of the epidermal growth factor receptor via an LL37-controlled mechanism [99].

Respiratory epithelial cells, alveolar macrophages, neutrophils, and mast cells all produce cathelicidin in the lungs [100,101]. Because the promoter region of LL37 contains a vitamin D response element, the production of LL37 in respiratory epithelial cells is regulated by a vitamin-D-dependent pathway [102]. The presence of consensus binding sites for NFkβ, IL-6, acute phase response factor, and IFN-γ response element in LL37 genes suggests that LL37 gene expression is regulated by these factors [35]. Different human cell types, including keratinocytes, monocytes, neutrophils, and bone-marrow-derived macrophages, have been demonstrated to regulate LL37 gene expression under the influence of vitamin D response elements [102]. TLR-mediated signals are found to control the vitamin D receptor and Cyp27B1 (an enzyme that catalyzes the conversion of 25-hydroxyvitamin D3 to the active 1,25-hydroxyvitamin D3). Furthermore, 1,25-hydroxyvitamin D3 is linked to increased CD14 and TLR-2 production. These findings pointed to a direct relationship between TLR activation, vitamin D receptor modulation, and LL37 gene regulation, all of which play a role in the immune response during infection [103].

Surfactant proteins (SP) are produced in the lungs and are either hydrophobic or hydrophilic. Their major job is to keep the surface tension of the air–liquid contact constant, preventing lung collapse and overinflation. SP also has antimicrobial, anti-inflammatory, immunomodulatory, and surfactant regulation functions [104,105]. As early as the alveolar stage, which is the final stage of lung formation, SP synthesis and secretion are regulated in tandem with the developmental process [106]. Surfactant proteins are regulated by hormones, growth factors, and other regulatory chemicals in addition to the lung developmental stage. Different variables govern the surfactants SP-A and SP-D, and investigations have suggested that they are regulated independently of one another. Overall, gene expression regulation of AMPs is tightly regulated throughout the respiratory tract along with intervened immunomodulatory activities.

### 3.1. Toll-like and Cytokine Receptors Mediate Regulation

Several bacterial stressors and inflammatory factors regulate the transcription of β-defensins in the respiratory tract. AMP expression in the respiratory tract is increased in response to infection and inflammation induced by a variety of respiratory pathogens [49]. Pathogen-associated molecular patterns (PAMPs) are directly detected by epithelial cells of the respiratory tract via a toll-like receptor (TLR)-mediated pathway, according to several studies. TLRs also regulates the expression of β-defensins—such as HBD-2 [107]. TLR2, TLR3, TLR4, TLR5, TLR6, and TLR9, have all been discovered to have a role in the expression of hBD2—by binding to their respective TLR ligands [108,109,110].

Defensins are influenced by cytokines and inflammatory mediators in addition to TLR signaling. TNF and IL-1β, which are generated by macrophages during infection and promote innate immune activities via TLRs in the respiratory tract, were found to induce hBD2 in the respiratory epithelium [94,111]. Additionally, the generation of cytokine IL-17 in the respiratory tract regulates the expression of β-defensins in Th17 cells [112]. The expression of hBD3 is increased by interferon stimulation, which is controlled by STAT1 and inhibited by IL-4 and IL-13 [113]. Evidence suggests that tight regulation of signaling pathways governs the production of β-defensins. TLR- and IL-17-dependent transcription control of HBD2 is further regulated by MAP kinases, transcription factor NFkB, and AP-1 activation according to reports [114]. In light of available reports, TLR- and cytokine receptor-mediated gene regulation of AMPs in the respiratory tract could be a potential therapeutic target to combat respiratory infections.

### 3.2. Vitamin-D-Dependent Regulation

Vitamin D3 is the biologically active form of vitamin D, and its immunomodulatory properties have long been known. Several investigations have also shown that vitamin D3 has HDP-inducing effects [115]. Direct treatment of respiratory epithelial cells with 1,25-dihydroxyvitamin D(3) has been observed to promote LL37 expression [116]. Vitamin D response element (VDRE) is found in both LL37 and HBD2 gene promoters, and vitamin D can directly activate their expression in the respiratory tract, however, in comparison to LL37, the reaction to hBD-2 expression is substantially weaker [102]. Furthermore, vitamin D can indirectly cause AMP release in the respiratory tract by increasing PAMP recognition, which is accomplished by TLR2 and CD14 upregulation [117,118]. In isolated human keratinocytes, monocytes, neutrophils, normal human bronchial epithelial cells, and myeloid cells, vitamin D3 has been demonstrated to stimulate gene expression of defensins and cathelicidins [102,119,120]. In the presence of muramyl dipeptide, vitamin D3 has been shown to stimulate gene expression of hBD2 and LL37 in primary human monocytes and epithelial cells [121]. Vitamin D3 has also been shown to increase LL37 gene expression and protect against bacterial infection in the urinary bladder [122]. In addition, calcitriol, a vitamin D active metabolite, is found to be effective in increasing LL37 gene expression in breast cancer cells [123]. Vitamin D3 has also been observed to promote hBD2 expression in peripheral mononuclear cells in humans, lowering infection rates [124]. Vitamin D3 has been suggested as a therapeutic method for bacterial infection and viral infections such as COVID-19 [125]. Additionally, vitamin D3 may be utilized to treat respiratory viral infections as a treatment option because HBD2 is known to be produced in the respiratory tract during viral infections. Conclusively, AMPs expression in the respiratory tract is tightly regulated with Vitamin D which suggested Vitamin D as a potential nutraceutical to prevent respiratory viral infections.

### 3.3. Signaling Pathways Involved in the Regulation of Defensins and Cathelicidins

MAPK signaling pathways are involved in a variety of biological activities, such as cellular differentiation, proliferation, and death, as well as the immune response during disease [126,127]. The inducible production of defensins and cathelicidins is similarly mediated by MAPK signaling pathways. Transactivation of the cathelicidin promoter occurred via three MAPK kinase cascades (ERK1/2, JNK, and p38 kinase) that regulate the transcription factor and activator protein-1, as shown by sodium-butyrate-induced production of LL37 in human lung epithelial cells [128,129]. Butyrate has been shown to induce LL37 transcription in human colonic epithelial cells via the MAPK-ERK pathway as well as LL37 expression in human intestinal epithelial cells via the MAPK-ERK and MAPK-p38 pathways [130,131]. Furthermore, phenylbutyrate has been shown to directly stimulate LL37 gene expression via the MAPK-ERK and JNK pathways [132]. In human primary keratinocytes, the MAPK-ERK signaling pathway regulates LL37 gene expression caused by lithocholic acid [133]. In Coco-2 cells, phosphorylation of ERK and activation of p38 MAP kinase are required for zinc-induced LL37 expression.

NFkB is involved in a toll-like-receptor-stimulated signaling cascade that regulates the expression of target genes and modulates the immune response [134]. The role of NFkB in HDP inducible gene expression has been widely documented and investigated in several research articles. In a study, it was discovered that in the presence of L-isoleucine, epithelial defensin expression in bovine kidney epithelial cells is stimulated by the activation of tans-activating factors of the NFkB [135]. Gene expression of hBD2 in human bronchial and pulmonary gland epithelial cells via the NFkB signaling pathway is stimulated by andrographolide, a plant diterpenoid, and paeoniflorin, a non-steroid anti-inflammatory medication. The MAPK-p38 and ERK singling pathways were also engaged in paeoniflorin-dependent activation of HBD2 [136,137]. Furthermore, the MAPK-p38 signaling pathway induces the gene expression of hBD2 in human lung epithelial and colon cancer cells by both andrographolide and dehydroandrographolide [136,138]. In two separate studies, resveratrol, a stilbenoid, and genistein, a dietary supplement, were also found to be involved in NFkB-c/EBP-α dependent gene expression induction of LL37 in human keratinocytes via stimulation of the sphingosine-1-phosphate (SIP) signaling pathway. Additionally, resveratrol induced LL37 gene expression in human keratinocytes via the MAPK-ERK pathway [139,140].

**Figure 1:**
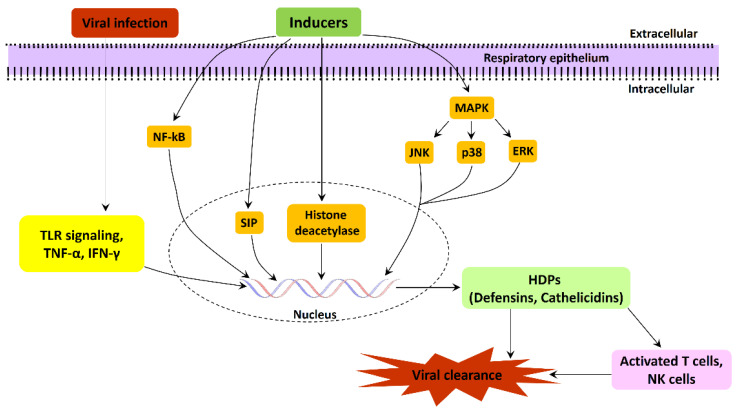
Schematic representation of various signaling pathways involved in the gene expression regulation of AMPs during respiratory tract viral infections.

HDPs are known to down-regulate gene transcription and associated gene expression regulation via histone deacetylases (HDACs), chromatin-modifying enzymes. Butyrate is a well-known HDAC inhibitor that has also been shown to stimulate the expression of endogenous pBD-2, pBD-3, protegrin 1–5, and myeloid antimicrobial peptide 36 in piglet macrophages [141]. Caprylic acid and nonanoic acid are likewise found to suppress the HDAC pathway, resulting in enhanced pBD-1 and pBD-2 gene expression [142]. In human lung epithelial cells, sodium butyrate induces LL37 expression by increasing the histone acetylation of an LL37 promoter [129]. The involvement of various signaling pathways for the gene expression regulation of AMPs in the respiratory tract suggested a multitier and tight regulation of AMPs expression and their role in protection against viral infections (Figure 1). These signaling networks could be a target for the development of inducible switches for AMPs, specifically during viral infection.

## 4. Nutrients Involved in the Regulation of Defensins and Cathelicidins

### 4.1. Amino Acids

Amino acids have been linked to the stimulation of immunological responses as well as the regulation of immune responses through lymphocyte proliferation and cytokine production [143]. Amino acids have previously been shown to induce the production of human defensin. L-isoleucine has been reported to stimulate the expression of human defensins in bovine kidney epithelial cells, swine jejunal epithelial cells, human peripheral blood mononuclear cells, and human colonic epithelial Caco-2 cells. However, because L-isoleucine required a stronger stimulation than D-isoleucine, the induction of expression is specific to confirmation [135,144]. Another study reported that L-arginine and L-isoleucine induced HBD1 expression but did not influence HBD2 expression in human colon cancer cells [145]. Following the findings, weaned pigs with 0.5 percent to 1.0 percent L-arginine supplementation showed increased gene expression of porcine β-defensins in the oral epithelium, tongue, ileum, and inguinal lymph nodes [146]. These findings suggested that essential amino acids have an essential role in defensin gene expression regulation. Next, dietary supplementation studies open up new avenues for the use of essential amino acids to increase the gene expression of HDPs. Furthermore, other than the regulation of defensin expression, basic amino acids can directly act against viruses. In a recent study, it was found that supplementation of lysine and reduction of arginine-rich food intake can ameliorate the infection caused by enveloped viruses such as SARS-CoV-2 and influenza [147]. Overall, amino acid supplementation could be used to induce specific HDPs in the respiratory tract to fight against viral infections in lung airways.

### 4.2. Fatty Acids and Analogs

The activation of gene expression of HDPs by fatty acids is known to alter the immune response and host immunity [148]. Short-chain fatty acids (SCFAs), acetate, propionate, and butyrate are colonic bacterial fermentation products that have been shown to have a role in HDP induction [144,149]. SCFAs, interestingly, greatly promote HDP expression and have been shown to improve host immunity, illness resistance, and the ability to regulate infectious diseases [148,150,151]. Acetate, propionate, and butyrate, which are found in fermented kiwifruit, have been shown to promote HBD1 and HBD2 expression in colonic epithelial cells [152].

LL37 is reported to be induced by fatty acids in addition to defensins. Through AP-1 and histone acetylation of the LL37 promoter, sodium butyrate has been shown to upregulate and stimulate LL37 gene expression in lung epithelial cells [129]. It has been found that isobutyrate, propionate, phenylbutyrate, isovaleric, isobutyric acids, valerate, hexanoate, and heptanoate all induce LL37 gene expression in human lung cells and colonic epithelial cells [130,132,153,154]. SCFAs were shown to have synergistic inducing effects on defensin and cathelicidin gene expression. However, combining phenylbutyrate with lactose was found to be substantially more effective in inducing LL37 gene expression in colonic epithelial cells [155]. Fatty acids’ ability to promote the gene expression of defensins and cathelicidin, as well as their potential application in the battle against respiratory viral infectious disease, was suggested by the data; nevertheless, more research and clinical studies are needed.

### 4.3. Carbohydrates and Conjugates

Carbohydrates are one of the most important energy-producing nutrients for humans. Glucose is a key carbohydrate and energy source that is also known to increase defensin and cathelicidin gene expression. Although studies have shown that glucose directly induces HBD1 and LL37 mRNA expression in human keratinocytes, renal cells, and embryonic kidney cells, high glucose concentrations reduced HBD mRNA expression and protein concentration in human keratinocytes [156,157,158,159]. Next, lactose as an immune inducer increased gene expression of LL37 in human breast milk, as demonstrated in colonic epithelial cells, monocytes, and macrophages [154,155]. Additionally, AV119, a natural avocado sugar, has likewise been shown to stimulate the expression of the HBD-2 gene in keratinocytes by activating protein kinase C, protein tyrosine kinase, and activator protein-1 [160,161]. Carbohydrates can also be utilized as a dietary supplement for the inducible expression of HDPs; however, further, studies are needed.

### 4.4. Plant Extracts

Plant extracts are known to have antiviral, antibacterial, antifungal, antioxidative, and immunomodulatory properties as well as the ability to maintain the gut microbiome [162,163]. Plant extracts have also been shown to influence gene expression as well as the gene expression induction of defensins and cathelicidins. A polyphenol called Epigallocatechin-3-gallate (EGCG) is found in many vegetables and fruits, including green tea, and has been shown to increase the release of defensins in gingival epithelial cells [164,165]. In gingival epithelial cells, green tea EGCG can promote the gene expression of both hBD1 and hBD2 [166]. In oral epithelial cells, a flavin derivative of black tea extract is reported to increase the gene expression of hBD1, hBD2, and hBD4 while lowering IL-8 production [167]. In a separate study, ellagic acid, a dimeric derivative of gallic acid found in a variety of fruits and vegetables, was found to stimulate hBD2 gene expression in human primary gingival epithelial cells [168]. In addition, Isatisindigotica root and plant-derived phenolic extracts are found to have anti-SARS-coronavirus-3C-like protease activity in a recent study [169]. According to reports, HDP-inducing plant extracts or chemicals could be used to treat bacterial and viral infections. However, further detailed research is needed.

### 4.5. Other Factors and Mechanisms

Other unclassified factors or substances, like defensins and cathelicidins, are known to activate the gene expression of HDPs in addition to the above-mentioned factors. Zinc, for example, raises the mRNA and, as a result, protein levels of porcine β-defensins 1, 2, and 3 in IPEC-J2 cells [170]. Calcium can also induce the gene expression of HBD2 and HBD3 in activated human keratinocytes [171]. Long-chain inulin-type fructans have also been shown to boost β-defensin-1 and LL37 expression in the mouse colon [172,173]. Lithocholic acid has also been shown to activate LL37 in colonic epithelial cells HT-29 [174]. In a separate investigation, aroylatedphenylenediamines are found to activate LL37 more efficiently in MN8CampLuc colonic epithelial cells when compared to butyrate and phenylbutyrate [175]. Additionally, some immunomodulatory medications, such as pimecrolimus, reported increasing LL37, hBD2, and hBD3 gene expression in human keratinocytes [176]. As a result of these findings, several HDP inducers may play an essential role in the fight against respiratory viral infectious diseases [154,177]. Furthermore, new cell-based assays could be developed and validated for high-throughput screening of novel inducers of LL37 and defensins disease control and prevention against respiratory tract viral infections.

The mTOR (mammalian target of rapamycin) and STAT3 signaling pathways have also been implicated in the up-regulation of -defensin gene expression by butyrate [178]. In a vitamin D receptor (VDR)-independent route, curcumin has been shown to promote LL37 expression in human monocytes [179]. Furthermore, bovine serum albumin has been demonstrated to promote hBD1 expression via a non-inflammatory mechanism requiring MYC proto-oncogene (c-myc) overexpression [145]. Furthermore, regulatory mechanisms involved in the expression of LL37 and activated by lactose and phenylbutyrate in colonic epithelial cells were found utilizing a proteomic method in a study [180].

## 5. Studying the Expression of HDPs and Protein–Protein Interaction (PPI) Networks Construction

**Figure 2:**
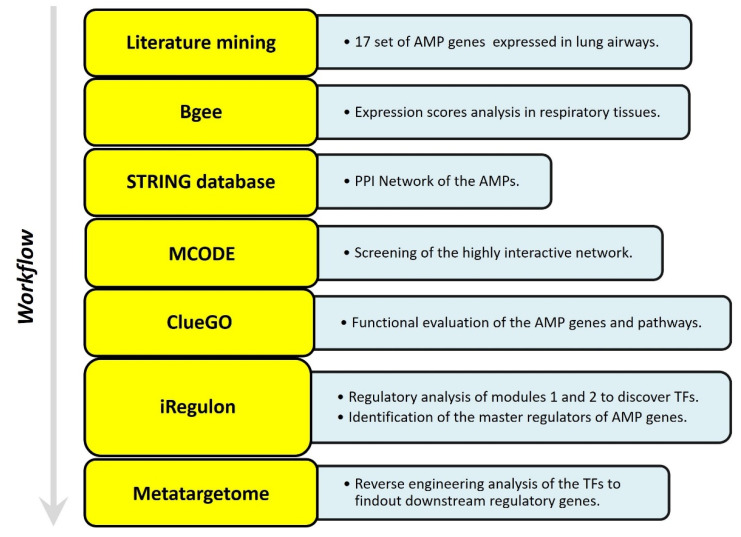
Adopted work-flow to study the gene expression profile of HDPs in lung airways habitat, and construction of PPI network.

The web-based gene expression database Bgee https://bgee.org/ (accessed on 5 January 2023) is employed for studying the expression levels of 17 HDPs as “expression scores” in various anatomical entities, from respiratory to gastrointestinal mucosa. The online database Search Tool for the Retrieval of Interacting Genes STRING, http://stringdb.org (accessed on 9 January 2023) is used to identify the interaction between the proteins and construct a PPI network. The molecular interaction network integrating gene expression profiles of HDPs in the respiratory tract is visualized using Cytoscape software (version 3.6.1). Furthermore, to identify the key modules in the PPI network the Cytoscape plugin MCODE (Molecular Complex Detection) is applied [181]. The degree cutoff was set as 5, and the rest of the parameters were set as default. Further, the plugin ClueGO is employed to analyze the pathway interaction network and annotate the function of key modules [182] (Figure 2).

### 5.1. Identifying the Transcription Factors and Validation

The transcription factors targeting the key modules were identified using the Cytoscape plugin iRegulon. The master regulators are those TFs whose target sets were found highly overlapping [183]. All the default parameters are left unchanged while predicting the TFs. Only those TFs are used to construct the regulatory network that covered more than 50% of genes and NES (Normalized Enrichment Score) > 5. Furthermore, reverse engineering is employed in metatargetome analysis to check the varied gene targets that overlap with our selected TFs. Expression levels of the TFs and target genes in various anatomical tissues were studied using the Bgee Gene expression Comparison webpage https://bgee.org/ (accessed on 5 January 2023). The results were displayed as maximum expression scores.

### 5.2. In Silico Analysis and Expression Profiling of Lung Airways Antimicrobial Peptides

**Figure 3:**
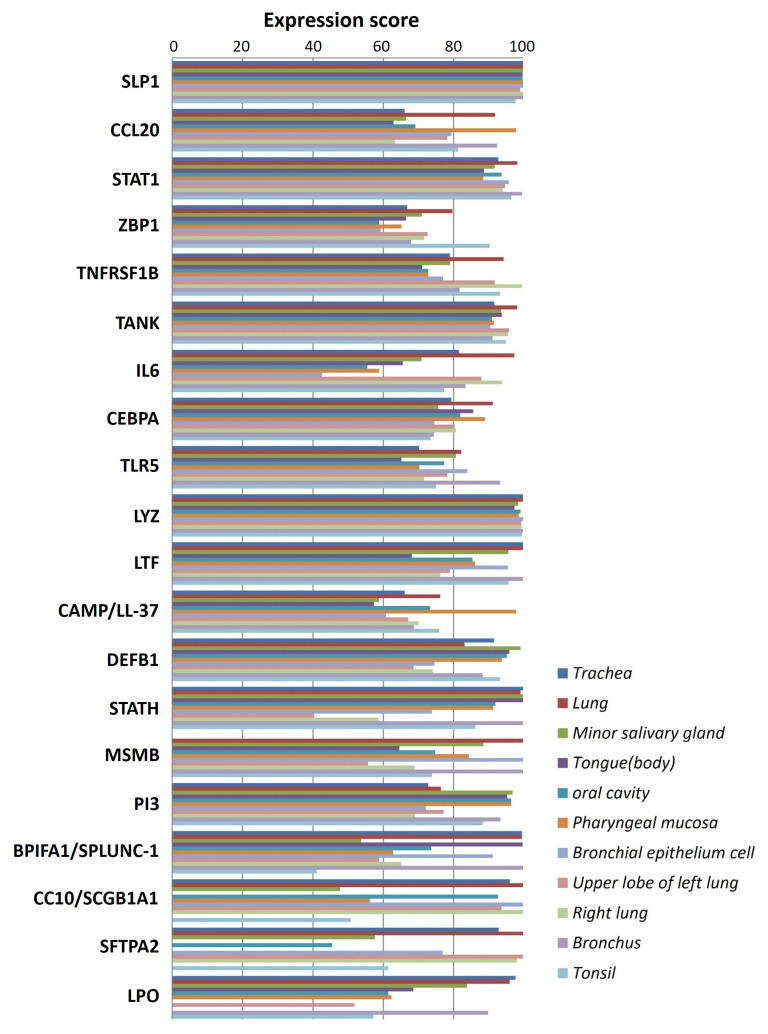
Antimicrobial peptide gene expression profile in lung airways. Expression levels of 20 potential genes responsible for antimicrobial activity in the lung airways/blood cells. The *Y*-axis shows the expression score represented by different color bars while the *X*-axis shows different genes expressed in respective tissue or blood cells in the respiratory tracts/lung airways.

**Figure 4:**
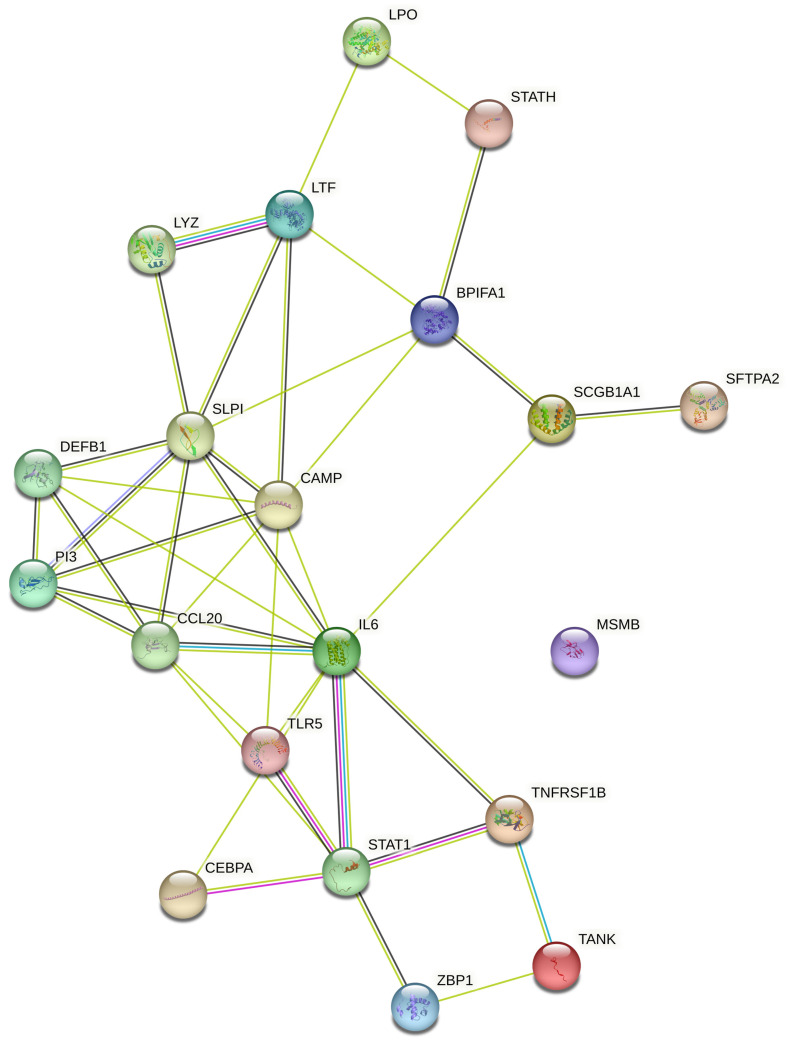
Protein–protein interaction network generated using STRING database. Different-colored circles represent the different antimicrobial peptides while connecting lines represent various interactions between the studied AMPs. Peptide shows more than one type of interaction connected with multiple lines respectively.

**Figure 5:**
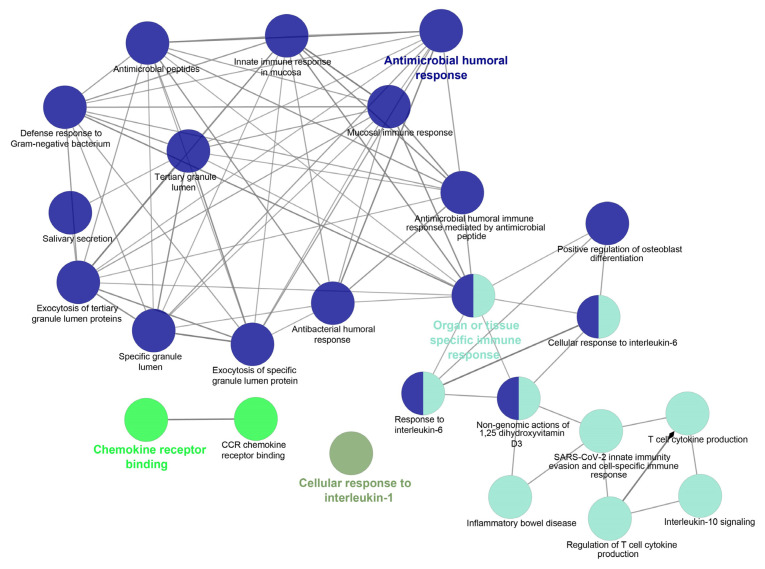
PPI Pathway analysis using Cytoscape plugin ClueGO to interpret the functional pathways and functions of each gene responsible for antimicrobial activity in lung airways. Different-colored circles indicate different functions or functional pathways as mentioned in the figure.

The literature mining revealed the 17 candidate genes (Table 2) responsible for antibacterial action in airways/blood cells. Using Bgee, a web-based gene expression database, the expression levels of all 20 genes in different organs are analyzed as “expression scores” (Figure 3). The expression scores are a quantitative terminology wherein the expression levels of each gene have been normalized on a scale and calculated. Antimicrobial genes have a significant expression in respiratory tissues, blood cells, and gastrointestinal entities, under varied conditions. The Bgee platform has accumulated all these expression data and represented them as expression scores. We used STRING to create a PPI network of all these known proteins (Figure 4) with PPI enrichment values < 1.0 × 10^−16^, indicating significantly strong interactions among themselves. Tissue enrichment detection analysis revealed significantly enriched tissues and body fluids including, mouth, salivary gland, pharynx, urine, and tears which corroborated with enrichment scores (using Bgee). The PPI network is then displayed using the Cytoscape plugin clueGO (Figure 5) to evaluate the functional pathways and functions of each gene involved in the antimicrobial activity in the lung airways. Furthermore, detailed PPI analysis found that the genes are engaged in a variety of pathways, including bacterial infection response, inflammatory response, negative regulation of the viral process, cytokine-mediated signaling pathway, cytokine production, cytokine receptor binding, chemokine receptor binding, cellular response to tumor necrosis factor, response to LPS, toll-like receptor signaling pathway, antifungal humoral response, TNF, regulation of T-cell cytokine production and SARS-Cov2 innate immune evasion and cell-specific immune response (Figure 5). STRING and culeGO study revealed a close physical and functional connection of antimicrobial genes in the lungs, which is implicated in a variety of cellular responses.

**Figure 6:**
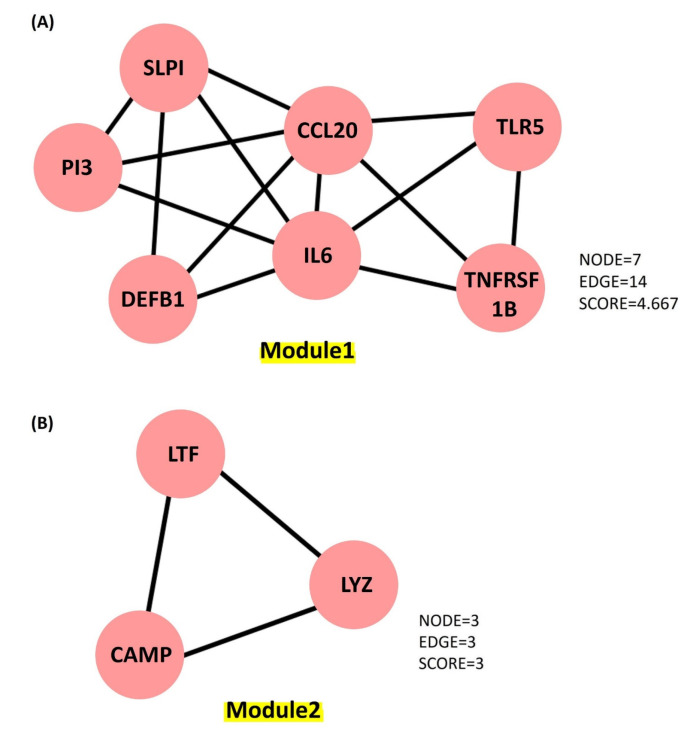
Key molecules identified from the PPI network generated using Cytoscape plugin MCOD. (**A**) Module 1 contains 7 proteins represented as 7 nodes and 14 edges, with a score of 4.667. (**B**) Module 2 contains 3 proteins represented as 3 nodes and 3 edges, with a score of 3.0.

The Cytoscape plugin Molecular Complex Detection (MCODE) is employed to study the highly dense interconnected clusters in the PPI network. Based on connectivity data and PPI analysis, two major modules with seven and three nodes were identified, respectively (Figure 6). Module 1 (seven genes) was discovered to be involved in defense response to bacteria, viral protein interactions with cytokines and cytokines receptors, the TNF signaling pathway, antimicrobial humoral responses, T-cell-mediated immunity, the TLR signaling system, interleukin-8 production, interleukin-10 signaling and calcium-driven signaling (Figure S1). Analysis for the genes present in Module 2 (threegenes) is discovered to be majorly involved in cellular responses to peptidoglycan, mucosal innate immunity, salivary production, gram-positive bacteria defense, amyloid precursor proteins from ordered fibrils and siderophore-dependent iron import into cells (Figure S2). In systems biology, identifying the master regulators of a biological pathway is a challenging aspect. We also used iRegulon to conduct regulatory analysis of both Modules 1 and 2 to discover transcription factors and master regulators involved in the expression control of the genes listed (Figure S3). Amongst several transcription factors (TFs) deduced from iRegulon analysis, only those with maximum targets and high Normalized Enrichment Scores (NES, >4.0) are chosen as the key regulators. The master regulators are considered to be transcription factors with significantly overlapping transcriptional target groups with the reported gene signatures (Figure S3A,B). We also used iRegulon to perform reverse engineering and metatargetome analysis on both Modules 1 and 2 using our selected TFs to visualize the other targets of the TFs and their interaction network (Figure S4A,B). The extended metatargetome analysis makes it clear that the overlapping targets (in the metatargetome network) indicate a core set of genes being regulated by these TFs.

The bioinformatic analysis of lung airways AMPs and their transcription regulator factors revealed the set of master regulators involved in specific designated cellular functions. By using the strategy and data presented in this study, one can predict the modulated HDPs in lung airways and their master regulators. The same strategy could be applied to respiratory infection conditions, which can reveal the possible HDP master regulators to be targeted to combat the diseases.

## 6. Conclusions

The present study explored the respiratory tract HDPs and their expression regulation in various cells like monocytes, macrophages, neutrophils, epithelial cells, keratinocytes, and mast cells during infection and host defense. The expression profile of 17 screened HDPs, responsible for antimicrobial activities in the various tissues and organs involved in the functional respiratory tract, has also been analyzed (Figure 2). Signaling pathways, such as MAPKs, NFkB, and histone acetylation, are involved in the gene expression regulation and induction of HDPs by several factors, such as amino acids, fatty acids, polyphenols, and vitamin D. Although in vitro studies suggested the role of immunomodulatory properties of HDPs during respiratory infections, in vivo studies for these properties of HDPs in the respiratory tract haven’t been conducted yet. Additionally, high-throughput screening assays have been developed to identify the multiple factors or compounds that can induce HDP gene expression [175,177]. Further dietary supplementation of HDP-inducing nutrients may serve as novel host-directed therapies or potential alternatives to treat respiratory diseases, such as COVID-19 [184,185,186,187]. The PPI network (Figure 3) and an elaboration of the pathways they govern (Figure 4) suggested that the genes are active participants in antimicrobial defense systems. Furthermore, amongst this interaction, there are two sets of highly interactive hubs constituting sets of 7 and 3 genes, respectively. Next, Figures S1–S4 confirm the participation of these two gene modules in defense against bacterial and viral infections.

Based on our review, along with expression and interaction network analysis, we suggested the importance of respiratory tract HDPs, their inducing factors, and TFs regulating individual pathways of antimicrobial defense as therapeutic agents to combat infectious diseases. However, further in-detail studies are required to explore the potential of HDPs, gene-expression-inducing factors, and TFs for protection against respiratory infections. This review is an outline to decipher the HDPs and their regulatory factors.

## 7. Limitations of the Study

The gene expression studies and PPI network analysis in the present study are solely based on the bioinformatic analysis of the publicly available databases. Additionally, all the gene expression analyses were performed using the gene expression profiling of a healthy individual, and further in-detail analysis is needed to compare the gene expression profile of AMPs in various respiratory tract infections. Furthermore, in vitro and in vivo studies are warranted to explore and fully understand the gene expression regulation of AMPs during different viral respiratory tract infections.

## Figures and Tables

**Table 1:** Major AMPs/factors produced in the lung and respiratory tract.

AMPs/Factors	Distribution in Lung	References
Defensins (α and β defensins 1–4)	Lung epithelium, airways, neutrophils	[17,18,19]
Cathelicidins (LL37)	Lung epithelium, airways, respiratory sub-mucosal glands, neutrophils	[20,21]
Surfactant protein-A and surfactant protein-D (SP-A, SP-D)	Lung epithelium	[22,23]
Lysozyme	Tracheobronchial sub-mucosal glands, tracheal and bronchial surface epithelium, neutrophils, alveolar macrophages, monocytes	[24,25]
Secretory leukocyte proteinase inhibitor (SLPI)	Respiratory sub-mucosal glands, monocytes, alveolar macrophages, neutrophils	[26,27]
Lactoferrin	Tracheobronchial sub-mucosal glands, tracheal surface epithelium, neutrophils	[24]
Phospholipase A2	Lung epithelium, neutrophils	[28,29]
Lactoperoxidase	Airways mucosal membrane	[30]
CCL20	Airways epithelium	[31]

**Table 2:** AMP genes, which are used in gene expression profiling.

Gene	Protein	Molecular Function (UniProt)	Ensemble ID
SLPI	Antileukoproteinase	Antibiotic, Antimicrobial, Protease inhibitor, Serine protease inhibitor	ENSG00000124107
CCL20	C-C Motif Chemokine 20	Antibiotic, Antimicrobial, Cytokine	ENSG00000115009
STAT 1	Signal transducer and activator of transcription 1-alpha/beta	Activator, DNA-binding	ENSG00000115415
ZBP1	Z-DNA-binding protein 1	DNA-binding	ENSG00000124256
TNFRSF1B	Tumor necrosis factor receptor superfamily member 1B	Receptor	ENSG00000028137
TANK	TRAF family member-associated NF-kappa-β activation	Ubiquitin protein ligase binding, metal ion binding.	ENSG00000136560
IL6	Interleukin-6	Cytokine, a Growth factor	ENSG00000136244
CEBPA	CCAAT/enhancer-binding protein alpha	Activator, Developmental protein, DNA-binding	ENSG00000245848
TLR5	Toll-like receptor 5	Receptor	ENSG00000187554
LTF	Lactotransferrin	Antibiotic, Antimicrobial, DNA-binding, Heparin-binding, Hydrolase, Protease, Serine protease	ENSG00000012223
LYZ	Lysozyme	Antimicrobial, Bacteriolytic enzyme, Glycosidase, Hydrolase, Milk protein	ENSG00000090382
LPO	Lactoperoxidase	Antimicrobial, Oxidoreductase, Peroxidase	ENSG00000167419
CAMP/LL37	Cathelicidin antimicrobial peptide	Antibiotic, Antimicrobial	ENSG00000164047
HBD-1	Beta-defensin 1	Antibiotic, Antimicrobial, Defensin	ENSG00000164825
CC10/CCSP/SCGB1A1A	Uteroglobin	Phospholipase A2 inhibitor	ENSG00000149021
STATH	Statgerin	Biomineralization	ENSG00000126549
MSMB	Beta-microseminoprotein	Secretion of mucus, antimicrobial andanti-inflammatory peptides	ENSG00000263639
BPIFA1	BPI fold containing family A member 1	Antibiotic, Antimicrobial	ENSG00000198183
PI3	Elafin	Kinase, Serine/threonine-protein kinase, Transferase	ENSG00000124102

## Data Availability

Not applicable.

## Supplementary Materials

The following supporting information can be downloaded at: https://www.mdpi.com/article/10.3390/clinpract13010012/s1. Figure S1: Functional annotation of genes of module 1 using Cytoscape Plugin ClueGO; Figure S2: Functional annotation of genes of module 2 using Cytoscape Plugin ClueGO; Figure S3: Regulatory and metatargetome analysis of module 1; Figure S4: Regulatory and metatargetome analysis of module 2.

## References

[1] Nicolas P. (2009). Multifunctional host defense peptides: Intracellular-targeting antimicrobial peptides. FEBS J..

[2] Nijnik A., Hancock R. (2009). Host defence peptides: Antimicrobial and immunomodulatory activity and potential applications for tackling antibiotic-resistant infections. Emerg. Heal. Threat. J..

[3] Drayton M., Deisinger J.P., Ludwig K.C., Raheem N., Müller A., Schneider T., Straus S.K. (2021). Host Defense Peptides: Dual Antimicrobial and Immunomodulatory Action. Int. J. Mol. Sci..

[4] Zhang L.J., Gallo R.L. (2016). Antimicrobial peptides. Curr. Biol..

[5] Robinson K., Deng Z., Hou Y., Zhang G. (2015). Regulation of the Intestinal Barrier Function by Host Defense Peptides. Front. Veter- Sci..

[6] Baindara P., Chakraborty R., Holliday Z., Mandal S., Schrum A. (2021). Oral probiotics in coronavirus disease 2019: Connecting the gut–lung axis to viral pathogenesis, inflammation, secondary infection and clinical trials. New Microbes New Infect..

[7] Manna S., Chowdhury T., Chakraborty R., Mandal S.M. (2020). Probiotics-Derived Peptides and Their Immunomodulatory Molecules Can Play a Preventive Role Against Viral Diseases Including COVID-19. Probiotics Antimicrob. Proteins.

[8] Haney E.F., Mansour S.C., Hancock R.E.W. (2017). Antimicrobial peptides: An introduction. Methods in Molecular Biology.

[9] Hilchie A.L., Wuerth K., Hancock R.E.W. (2013). Immune modulation by multifaceted cationic host defense (antimicrobial) peptides. Nat. Chem. Biol..

[10] Manna S., Baindara P., Mandal S.M. (2020). Molecular pathogenesis of secondary bacterial infection associated to viral infections including SARS-CoV-2. J. Infect. Public Health.

[11] Rogan M.P., Geraghty P., Greene C.M., O’Neill S.J., Taggart C.C., McElvaney N.G. (2006). Antimicrobial proteins and polypeptides in pulmonary innate defence. Respir. Res..

[12] Mookherjee N., Anderson M.A., Haagsman H.P., Davidson D.J. (2020). Antimicrobial host defence peptides: Functions and clinical potential. Nat. Rev. Drug Discov..

[13] Beisswenger C., Bals R. (2005). Antimicrobial Peptides in Lung Inflammation. Chem. Immunol. Allergy.

[14] Lai Y.P., Gallo R.L. (2009). AMPed up immunity: How antimicrobial peptides have multiple roles in immune defense. Trends Immunol..

[15] Manna P.B.A.S.M.M.S., Chowdhury T., Baindara P., Mandal S.M. (2020). Fusion Protein Targeted Antiviral Peptides: Fragment-Based Drug Design (FBDD) Guided Rational Design of Dipeptides Against SARS-CoV-2. Curr. Protein Pept. Sci..

[16] Baindara P., Roy D., Mandal S.M., Schrum A.G. (2022). Conservation and Enhanced Binding of SARS-CoV-2 Omicron Spike Protein to Coreceptor Neuropilin-1 Predicted by Docking Analysis. Infect. Dis. Rep..

[17] McCray P.B., Bentley L. (1997). Human airway epithelia express a beta-defensin. Am. J. Respir Cell Mol. Biol..

[18] Diamond G., Legarda D., Ryan L.K. (2000). The innate immune response of the respiratory epithelium. Immunol. Rev..

[19] Schutte B.C., McCray P.B. (2002). β-Defensins in Lung Host Defense. Annu. Rev. Physiol..

[20] Bals R., Wang X., Zasloff M., Wilson J.M. (1998). The peptide antibiotic ll-37/hcap-18 is expressed in epithelia of the human lung where it has broad antimicrobial activity at the airway surface. Proc. Natl. Acad. Sci. USA.

[21] Zanetti M. (2004). Cathelicidins, multifunctional peptides of the innate immunity. J. Leukoc. Biol..

[22] Crouch E., Parghi D., Kuan S.F., Persson A. (1992). Surfactant protein D: Subcellular localization in nonciliated bronchiolar epithelial cells. Am. J. Physiol. Cell. Mol. Physiol..

[23] Kuroki Y., Takahashi M., Nishitani C. (2007). Pulmonary collectins in innate immunity of the lung. Cell. Microbiol..

[24] Dubin R.F., Robinson S.K., Widdicombe J.H. (2004). Secretion of lactoferrin and lysozyme by cultures of human airway epithelium. Am. J. Physiol. Cell. Mol. Physiol..

[25] Ganz T. (2003). Antimicrobial polypeptides. J. Leukoc. Biol..

[26] Sallenave J.M. (2002). Antimicrobial activity of antiproteinases. Biochem. Soc. Trans..

[27] Moraes T.J., Chow C.W., Downey G.P. (2003). Proteases and lung injury. Crit. Care Med..

[28] Lindbom J., Ljungman A.G., Lindahl M., Tagesson C. (2002). Increased Gene Expression of Novel Cytosolic and Secretory Phospholipase A_2_Types in Human Airway Epithelial Cells Induced by Tumor Necrosis Factor-*α* and IFN-*γ*. J. Interf. Cytokine Res..

[29] Gimenez A.P., Wu Y.-Z., Paya M., Delclaux C., Touqui L., Goossens P.L. (2004). High Bactericidal Efficiency of Type IIA Phospholipase A2 against *Bacillus anthracis* and Inhibition of Its Secretion by the Lethal Toxin. J. Immunol..

[30] Christensen T.G., Blanchard G.C., Nolley G., Hayes J.A. (1981). Ultrastructural localization of endogenous peroxidase in the lower respiratory tract of the guinea pig. Cell Tissue Res..

[31] Starner T.D., Barker C.K., Jia H.P., Kang Y., McCray P.B. (2003). CCL20 Is an Inducible Product of Human Airway Epithelia with Innate Immune Properties. Am. J. Respir. Cell Mol. Biol..

[32] Sørensen O.E., Thapa D.R., Rosenthal A., Liu L., Roberts A.A., Ganz T. (2005). Differential Regulation of β-Defensin Expression in Human Skin by Microbial Stimuli. J. Immunol..

[33] Shestakova T., Zhuravel E., Bolgova L., Alekseenko O., Soldatkina M., Pogrebnoy P. (2008). Expression of human beta-defensins-1, 2 and 4 mRNA in human lung tumor tissue: A pilot study. Exp. Oncol..

[34] Otte J.-M., Neumann H.M., Brand S., Schrader H., Schmidt W.E., Schmitz F. (2009). Expression of beta-defensin 4 is increased in human gastritis. Eur. J. Clin. Investig..

[35] Yang D., Biragyn A., Hoover D.M., Lubkowski J., Oppenheim J.J. (2004). Multiple Roles of Antimicrobial Defensins, Cathelicidins, and Eosinophil-Derived Neurotoxin in Host Defense. Annu. Rev. Immunol..

[36] Brown K.L., Hancock R.E. (2006). Cationic host defense (antimicrobial) peptides. Curr. Opin. Immunol..

[37] Yin L., Chino T., Horst O.V., Hacker B.M., Clark E.A., Dale B.A., Chung W.O. (2010). Differential and coordinated expression of defensins and cytokines by gingival epithelial cells and dendritic cells in response to oral bacteria. BMC Immunol..

[38] Xu C., Wang A., Marin M., Honnen W., Ramasamy S., Porter E., Subbian S., Pinter A., Melikyan G., Lu W. (2021). Human Defensins Inhibit SARS-CoV-2 Infection by Blocking Viral Entry. Viruses.

[39] Wang C., Wang S., Li D., Wei D.-Q., Zhao J., Wang J. (2020). Human Intestinal Defensin 5 Inhibits SARS-CoV-2 Invasion by Cloaking ACE2. Gastroenterology.

[40] Kudryashova E., Zani A., Vilmen G., Sharma A., Lu W., Yount J.S., Kudryashov D.S. (2022). Inhibition of SARS-CoV-2 Infection by Human Defensin HNP1 and Retrocyclin RC-101. J. Mol. Biol..

[41] Holly M.K., Diaz K., Smith J.G. (2017). Defensins in Viral Infection and Pathogenesis. Annu. Rev. Virol..

[42] Matsumura T., Sugiyama N., Murayama A., Yamada N., Shiina M., Asabe S., Wakita T., Imawari M., Kato T. (2015). Antimicrobial peptide LL-37 attenuates infection of hepatitis C virus. Hepatol. Res..

[43] Agerberth B., Gunne H., Odeberg J., Kogner P., Boman H.G., Gudmundsson G.H. (1995). FALL-39, a putative human peptide antibiotic, is cysteine-free and expressed in bone marrow and testis. Proc. Natl. Acad. Sci. USA.

[44] Harcourt J.L., McDonald M., Svoboda P., Pohl J., Tatti K., Haynes L.M. (2016). Human cathelicidin, LL-37, inhibits respiratory syncytial virus infection in polarized airway epithelial cells. BMC Res. Notes.

[45] Agier J., Efenberger M., Brzezińska-Błaszczyk E. (2015). Review paper Cathelicidin impact on inflammatory cells. Central Eur. J. Immunol..

[46] Bandurska K., Berdowska A., Barczyńska-Felusiak R., Krupa P. (2015). Unique features of human cathelicidin LL-37. Biofactors.

[47] Sousa F.H., Casanova V., Findlay F., Stevens C., Svoboda P., Pohl J., Proudfoot L., Barlow P.G. (2017). Cathelicidins display conserved direct antiviral activity towards rhinovirus. Peptides.

[48] Currie S.M., Findlay E.G., McFarlane A.J., Fitch P.M., Böttcher B., Colegrave N., Paras A., Jozwik A., Chiu C., Schwarze J. (2016). Cathelicidins Have Direct Antiviral Activity against Respiratory Syncytial Virus In Vitro and Protective Function In Vivo in Mice and Humans. J. Immunol..

[49] Parker D., Prince A. (2011). Innate Immunity in the Respiratory Epithelium. Am. J. Respir. Cell Mol. Biol..

[50] Telang S. (2018). Lactoferrin: A Critical Player in Neonatal Host Defense. Nutrients.

[51] Travis S.M., Conway B.-A.D., Zabner J., Smith J.J., Anderson N.N., Singh P.K., Greenberg E.P., Welsh M.J. (1999). Activity of Abundant Antimicrobials of the Human Airway. Am. J. Respir. Cell Mol. Biol..

[52] Singh P.K., Tack B.F., McCray P., Welsh M. (2000). Synergistic and additive killing by antimicrobial factors found in human airway surface liquid. Am. J. Physiol. Cell. Mol. Physiol..

[53] Berlutti F., Pantanella F., Natalizi T., Frioni A., Paesano R., Polimeni A., Valenti P. (2011). Antiviral Properties of Lactoferrin—A Natural Immunity Molecule. Molecules.

[54] Hirashima N., Orito E., Ohba K., Kondo H., Sakamoto T., Matsunaga S., Kato A., Nukaya H., Sakakibara K., Ohno T. (2004). A randomized controlled trial of consensus interferon with or without lactoferrin for chronic hepatitis C patients with genotype 1b and high viral load. Hepatol. Res..

[55] Shin K., Wakabayashi H., Yamauchi K., Teraguchi S., Tamura Y., Kurokawa M., Shiraki K. (2005). Effects of orally administered bovine lactoferrin and lactoperoxidase on influenza virus infection in mice. J. Med Microbiol..

[56] Elass E., Masson M., Mazurier J., Legrand D. (2002). Lactoferrin Inhibits the Lipopolysaccharide-Induced Expression and Proteoglycan-Binding Ability of Interleukin-8 in Human Endothelial Cells. Infect. Immun..

[57] Wakabayashi H., Oda H., Yamauchi K., Abe F. (2014). Lactoferrin for prevention of common viral infections. J. Infect. Chemother..

[58] Kell D.B., Heyden E.L., Pretorius E. (2020). The Biology of Lactoferrin, an Iron-Binding Protein That Can Help Defend Against Viruses and Bacteria. Front. Immunol..

[59] Berthon B.S., Williams L.M., Williams E.J., Wood L.G. (2022). Effect of Lactoferrin Supplementation on Inflammation, Immune Function, and Prevention of Respiratory Tract Infections in Humans: A Systematic Review and Meta-analysis. Adv. Nutr. Int. Rev. J..

[60] Ali A.S., Hasan S.S., Know C.S., Merchant H.A. (2021). Lactoferrin reduces the risk of respiratory tract infections: A meta-analysis of randomized controlled trials. Clin. Nutr. ESPEN.

[61] Kouchi I., Yasuoka S., Ueda Y., Ogura T. (1993). Analysis of secretory leukocyte protease inhibitor (SLPI) in bronchial secretions from patients with hypersecretory respiratory diseases. Tokushima J. Exp. Med..

[62] Majchrzak-Gorecka M., Majewski P., Grygier B., Murzyn K., Cichy J. (2016). Secretory leukocyte protease inhibitor (SLPI), a multifunctional protein in the host defense response. Cytokine Growth Factor Rev..

[63] Weldon S., McNally P., McElvaney N.G., Elborn J.S., McAuley D.F., Wartelle J., Belaaouaj A., Levine R.L., Taggart C.C. (2009). Decreased Levels of Secretory Leucoprotease Inhibitor in the *Pseudomonas*-Infected Cystic Fibrosis Lung Are Due to Neutrophil Elastase Degradation. J. Immunol..

[64] Hiemstra P.S., Maassen R.J., Stolk J., Heinzel-Wieland R., Steffens G.J., Dijkman J.H. (1996). Antibacterial activity of antileukoprotease. Infect. Immun..

[65] Lentsch A.B., Jordan J.A., Czermak B.J., Diehl K.M., Younkin E.M., Sarma V., Ward P.A. (1999). Inhibition of NF-κB Activation and Augmentation of IκBβ by Secretory Leukocyte Protease Inhibitor during Lung Inflammation. Am. J. Pathol..

[66] Lentsch A.B., Yoshidome H., Warner R.L., Ward P.A., Edwards M.J. (1999). Secretory leukocyte protease inhibitor in mice regulates local and remote organ inflammatory injury induced by hepatic ischemia/reperfusion. Gastroenterology.

[67] Greene C., Taggart C., Lowe G., Gallagher P., McElvaney N., O’Neill S. (2003). Local Impairment of Anti–Neutrophil Elastase Capacity in Community-Acquired Pneumonia. J. Infect. Dis..

[68] Meyer M., Kesic M.J., Clarke J., Ho E., Simmen R.C., Diaz-Sanchez D., Noah T.L., Jaspers I. (2013). Sulforaphane induces SLPI secretion in the nasal mucosa. Respir. Med..

[69] Ganz T. (2006). Lysozyme. Encyclopedia of Respiratory Medicine, Four-Volume Set.

[70] Akinbi H.T., Epaud R., Bhatt H., Weaver T.E. (2000). Bacterial Killing Is Enhanced by Expression of Lysozyme in the Lungs of Transgenic Mice. J. Immunol..

[71] Dajani R., Zhang Y., Taft P.J., Travis S.M., Starner T.D., Olsen A., Zabner J., Welsh M.J., Engelhardt J.F. (2005). Lysozyme Secretion by Submucosal Glands Protects the Airway from Bacterial Infection. Am. J. Respir. Cell Mol. Biol..

[72] Song Y., Zhang H., Zhu Y., Zhao X., Lei Y., Zhou W., Yu J., Dong X., Wang X., Du M. (2022). Lysozyme Protects Against Severe Acute Respiratory Syndrome Coronavirus 2 Infection and Inflammation in Human Corneal Epithelial Cells. Investig. Opthalmology Vis. Sci..

[73] Brunaugh A.D., Seo H., Warnken Z., Ding L., Seo S.H., Smyth H.D.C. (2021). Development and evaluation of inhalable composite niclosamide-lysozyme particles: A broad-spectrum, patient-adaptable treatment for coronavirus infections and sequalae. PLoS ONE.

[74] Jiang L., Li Y., Wang L., Guo J., Liu W., Meng G., Zhang L., Li M., Cong L., Sun M. (2021). Recent Insights Into the Prognostic and Therapeutic Applications of Lysozymes. Front. Pharmacol..

[75] Wijkstrom-Frei C., El-Chemaly S., Ali-Rachedi R., Gerson C., Cobas M.A., Forteza R., Salathe M., Conner G.E. (2003). Lactoperoxidase and Human Airway Host Defense. Am. J. Respir. Cell Mol. Biol..

[76] Fischer A.J., Lennemann N.J., Krishnamurthy S., Pócza P., Durairaj L., Launspach J.L., Rhein B.A., Wohlford-Lenane C., Lorentzen D., Bánfi B. (2011). Enhancement of Respiratory Mucosal Antiviral Defenses by the Oxidation of Iodide. Am. J. Respir. Cell Mol. Biol..

[77] Patel U., Gingerich A., Widman L., Sarr D., Tripp R.A., Rada B. (2018). Susceptibility of influenza viruses to hypothiocyanite and hypoiodite produced by lactoperoxidase in a cell-free system. PLoS ONE.

[78] Homey B., Dieu-Nosjean M.-C., Wiesenborn A., Massacrier C., Pin J.-J., Oldham E., Catron D., Buchanan M.E., Müller A., deWaal Malefyt R. (2000). Up-Regulation of Macrophage Inflammatory Protein-3α/CCL20 and CC Chemokine Receptor 6 in Psoriasis. J. Immunol..

[79] Harant H., Eldershaw S.A., Lindley I.J. (2001). Human macrophage inflammatory protein-3α/CCL20/LARC/Exodus/SCYA20 is transcriptionally upregulated by tumor necrosis factor-α via a non-standard NF-κB site. FEBS Lett..

[80] Ghosh M., Shen Z., Schaefer T.M., Fahey J.V., Gupta P., Wira C.R. (2009). ORIGINAL ARTICLE: CCL20/MIP3α is a Novel Anti-HIV-1 Molecule of the Human Female Reproductive Tract. Am. J. Reprod. Immunol..

[81] Khalil B.A., Elemam N.M., Maghazachi A.A. (2021). Chemokines and chemokine receptors during COVID-19 infection. Comput. Struct. Biotechnol. J..

[82] Selsted M.E., Ouellette A.J. (2005). Mammalian defensins in the antimicrobial immune response. Nat. Immunol..

[83] Frye M., Bargon J., Dauletbaev N., Weber A., Wagner T.O.F., Gropp R. (2000). Expression of human alpha-defensin 5 (HD5) mRNA in nasal and bronchial epithelial cells. J. Clin. Pathol..

[84] Kao C.Y., Chen Y., Zhao Y.H., Wu R. (2003). ORFeome-Based Search of Airway Epithelial Cell-Specific Novel Human β-Defensin Genes. Am. J. Respir. Cell Mol. Biol..

[85] Yanagi S., Ashitani J.-I., Ishimoto H., Date Y., Mukae H., Chino N., Nakazato M. (2005). Isolation of human β-defensin-4 in lung tissue and its increase in lower respiratory tract infection. Respir. Res..

[86] Harder J., Bartels J., Christophers E., Schröder J.-M. (2001). Isolation and Characterization of Human μ-Defensin-3, a Novel Human Inducible Peptide Antibiotic. J. Biol. Chem..

[87] Littmann M., Albiger B., Frentzen A., Normark S., Henriques-Normark B., Plant L. (2009). *Streptococcus pneumoniae* evades human dendritic cell surveillance by pneumolysin expression. EMBO Mol. Med..

[88] Benincasa M., Mattiuzzo M., Herasimenka Y., Cescutti P., Rizzo R., Gennaro R. (2009). Activity of antimicrobial peptides in the presence of polysaccharides produced by pulmonary pathogens. J. Pept. Sci..

[89] Scharf S., Vardarova K., Lang F., Schmeck B., Opitz B., Flieger A., Heuner K., Hippenstiel S., Suttorp N., N’Guessan P.D. (2010). Legionella pneumophila induces human beta Defensin-3 in pulmonary cells. Respir. Res..

[90] Liu L., Wang L., Jia H.P., Zhao C., Heng H.H., Schutte B.C., McCray P., Ganz T. (1998). Structure and mapping of the human β-defensin HBD-2 gene and its expression at sites of inflammation. Gene.

[91] Koczulla A.R., Bals R. (2003). Antimicrobial peptides: Current status and therapeutic potential. Drugs.

[92] Jia H.P., Schutte B.C., Schudy A., Linzmeier R., Guthmiller J.M., Johnson G.K., Tack B.F., Mitros J.P., Rosenthal A., Ganz T. (2001). Discovery of new human β-defensins using a genomics-based approach. Gene.

[93] Hancock R.E., Diamond G. (2000). The role of cationic antimicrobial peptides in innate host defences. Trends Microbiol..

[94] Harder J., Meyer-Hoffert U., Teran L.M., Schwichtenberg L., Bartels J., Maune S., Schröder J.-M. (2000). Mucoid *Pseudomonas aeruginosa*, TNF- α, and IL-1 β, but Not IL-6, Induce Human β -Defensin-2 in Respiratory Epithelia. Am. J. Respir. Cell Mol. Biol..

[95] Scott M.G., Vreugdenhil A.C.E., Buurman W.A., Hancock R.E.W., Gold M.R. (2000). Cutting Edge: Cationic Antimicrobial Peptides Block the Binding of Lipopolysaccharide (LPS) to LPS Binding Protein. J. Immunol..

[96] Scott M.G., Hancock R.E.W. (2000). Cationic antimicrobial peptides and their multifunctional role in the immune system. Crit. Rev. Immunol..

[97] Harder R., Meyer-Hoffert U., Wehkamp K. (2004). Differential Gene Induction of Human β-Defensins (hBD-1, -2, -3, and -4) in Keratinocytes Is Inhibited by Retinoic Acid. J. Investig. Dermatol..

[98] Van Es J.H., Jay P., Gregorieff A., Van Gijn M.E., Jonkheer S., Hatzis P., Thiele A., van den Born M., Begthel H., Brabletz T. (2005). Wnt signalling induces maturation of Paneth cells in intestinal crypts. Nat. Cell Biol..

[99] Sørensen O.E., Thapa D.R., Roupé K.M., Valore E.V., Sjöbring U., Roberts A.A., Schmidtchen A., Ganz T. (2006). Injury-induced innate immune response in human skin mediated by transactivation of the epidermal growth factor receptor. J. Clin. Investig..

[100] Rivas-Santiago B., Hernandez-Pando R., Carranza C., Juarez E., Contreras J.L., Aguilar-Leon D., Torres M., Sada E. (2008). Expression of Cathelicidin LL-37 during *Mycobacterium tuberculosis* Infection in Human Alveolar Macrophages, Monocytes, Neutrophils, and Epithelial Cells. Infect. Immun..

[101] Di Nardo A., Vitiello A., Gallo R.L. (2003). Cutting Edge: Mast Cell Antimicrobial Activity Is Mediated by Expression of Cathelicidin Antimicrobial Peptide. J. Immunol..

[102] Wang T.-T., Nestel F.P., Bourdeau V., Nagai Y., Wang Q., Liao J., Tavera-Mendoza L., Lin R., Hanrahan J.W., Mader S. (2004). Cutting Edge: 1,25-Dihydroxyvitamin D3 Is a Direct Inducer of Antimicrobial Peptide Gene Expression. J. Immunol..

[103] Liu P.T., Stenger S., Li H., Wenzel L., Tan B.H., Krutzik S.R., Ochoa M.T., Schauber J., Wu K., Meinken C. (2006). Toll-Like Receptor Triggering of a Vitamin D-Mediated Human Antimicrobial Response. Science.

[104] Han S., Mallampalli R.K. (2015). The Role of Surfactant in Lung Disease and Host Defense against Pulmonary Infections. Ann. Am. Thorac. Soc..

[105] Wright J.R. (2005). Immunoregulatory functions of surfactant proteins. Nat. Rev. Immunol..

[106] King R.J., Ruch J., Gikas E.G., Platzker A.C., Creasy R.K. (1975). Appearance of paoproteins of pulmonary surfactant in human amniotic fluid. J. Appl. Physiol..

[107] Tomita T., Nagase T., Ohga E., Yamaguchi Y., Yoshizumi M., Ouchi Y. (2002). Molecular mechanisms underlying human beta-defensin-2 gene expression in a human airway cell line (LC2/ad). Respirology.

[108] Hertz C.J., Wu Q., Porter E.M., Zhang Y.J., Weismüller K.-H., Godowski P.J., Ganz T., Randell S.H., Modlin R.L. (2003). Activation of Toll-Like Receptor 2 on Human Tracheobronchial Epithelial Cells Induces the Antimicrobial Peptide Human β Defensin-2. J. Immunol..

[109] MacRedmond R., Greene C., Taggart C.C., McElvaney N., O’Neill S. (2005). Respiratory epithelial cells require Toll-like receptor 4 for induction of Human β-defensin 2 by Lipopolysaccharide. Respir. Res..

[110] Froy O. (2005). Regulation of mammalian defensin expression by Toll-like receptor-dependent and independent signalling pathways. Cell. Microbiol..

[111] Hess C., Herr C., Beisswenger C., Zakharkina T., Schmid R.M., Bals R. (2009). Myeloid RelA regulates pulmonary host defense networks. Eur. Respir. J..

[112] Kao C.-Y., Chen Y., Thai P., Wachi S., Huang F., Kim C., Harper R.W., Wu R. (2004). IL-17 Markedly Up-Regulates β-Defensin-2 Expression in Human Airway Epithelium via JAK and NF-κB Signaling Pathways. J. Immunol..

[113] Albanesi C., Fairchild H.R., Madonna S., Scarponi C., De Pità O., Leung D.Y.M., Howell M.D. (2007). IL-4 and IL-13 Negatively Regulate TNF-α- and IFN-γ-Induced β-Defensin Expression through STAT-6, Suppressor of Cytokine Signaling (SOCS)-1, and SOCS-3. J. Immunol..

[114] Scharf S., Hippenstiel S., Flieger A., Suttorp N., N’Guessan P.D. (2010). Induction of human β-defensin-2 in pulmonary epithelial cells by *Legionella pneumophila*: Involvement of TLR2 and TLR5, p38 MAPK, JNK, NF-κB, and AP-1. Am. J. Physiol. Cell. Mol. Physiol..

[115] van der Does A.M., Kenne E., Koppelaar E., Agerberth B., Lindbom L. (2014). Vitamin D_3_ and phenylbutyrate promote development of a human dendritic cell subset displaying enhanced antimicrobial properties. J. Leukoc. Biol..

[116] Yim S., Dhawan P., Ragunath C., Christakos S., Diamond G. (2007). Induction of cathelicidin in normal and CF bronchial epithelial cells by 1,25-dihydroxyvitamin D3. J. Cyst. Fibros..

[117] Schauber J., Dorschner R.A., Coda A.B., Büchau A.S., Liu P.T., Kiken D., Helfrich Y.R., Kang S., Elalieh H.Z., Steinmeyer A. (2007). Injury enhances TLR2 function and antimicrobial peptide expression through a vitamin D–dependent mechanism. J. Clin. Investig..

[118] Schauber J., Oda Y., Büchau A.S., Yun Q.-C., Steinmeyer A., Zügel U., Bikle D.D., Gallo R.L. (2008). Histone Acetylation in Keratinocytes Enables Control of the Expression of Cathelicidin and CD14 by 1,25-Dihydroxyvitamin D3. J. Investig. Dermatol..

[119] Dhawan P., Wei R., Sun C., Gombart A.F., Koeffler H.P., Diamond G., Christakos S. (2014). C/EBPα and the Vitamin D Receptor Cooperate in the Regulation of Cathelicidin in Lung Epithelial Cells. J. Cell. Physiol..

[120] Gombart A.F., Borregaard N., Koeffler H.P. (2005). Human cathelicidin antimicrobial peptide (CAMP) gene is a direct target of the vitamin D receptor and is strongly up-regulated in myeloid cells by 1,25-dihydroxyvitamin D_3_. FASEB J..

[121] Wang T.-T., Dabbas B., Laperriere D., Bitton A.J., Soualhine H., Tavera-Mendoza L.E., Dionne S., Servant M.J., Bitton A., Seidman E.G. (2010). Direct and Indirect Induction by 1,25-Dihydroxyvitamin D3 of the NOD2/CARD15-Defensin β2 Innate Immune Pathway Defective in Crohn Disease. J. Biol. Chem..

[122] Hertting O., Holm Å., Lüthje P., Brauner H., Dyrdak R., Jonasson A.F., Wiklund P., Chromek M., Brauner A. (2010). Vitamin D Induction of the Human Antimicrobial Peptide Cathelicidin in the Urinary Bladder. PLoS ONE.

[123] García-Quiroz J., García-Becerra R., Santos-Martínez N., Avila E., Larrea F., Díaz L. (2016). Calcitriol stimulates gene expression of cathelicidin antimicrobial peptide in breast cancer cells with different phenotype. J. Biomed. Sci..

[124] Castañeda-Delgado J.E., Araujo Z., Gonzalez-Curiel I., Serrano C.J., Santiago C.R., Enciso-Moreno J.A., Rivas-Santiago B., Information R. (2016). Vitamin D and L-Isoleucine Promote Antimicrobial Peptide hBD-2 Production in Peripheral Blood Mononuclear Cells from Elderly Individuals. Int. J. Vitam. Nutr. Res..

[125] Paria K., Paul D., Chowdhury T., Pyne S., Chakraborty R., Mandal S.M. (2020). Synergy of melanin and vitamin-D may play a fundamental role in preventing SARS-CoV-2 infections and halt COVID-19 by inactivating furin protease. Transl. Med. Commun..

[126] Wancket L.M., Frazier W.J., Liu Y. (2012). Mitogen-activated protein kinase phosphatase (MKP)-1 in immunology, physiology, and disease. Life Sci..

[127] Mandal S.M., Manna S., Mondal S., Ghosh A.K., Chakraborty R. (2018). Transcriptional regulation of human defense peptides: A new direction in infection control. Biol. Chem..

[128] Robinson K., Ma X., Liu Y., Qiao S., Hou Y., Zhang G. (2018). Dietary modulation of endogenous host defense peptide synthesis as an alternative approach to in-feed antibiotics. Anim. Nutr..

[129] Kida Y., Shimizu T., Kuwano K. (2006). Sodium butyrate up-regulates cathelicidin gene expression via activator protein-1 and histone acetylation at the promoter region in a human lung epithelial cell line, EBC-1. Mol. Immunol..

[130] Schauber J., Svanholm C., Termén S., Iffland K., Menzel T., Scheppach W., Melcher R., Agerberth B., Lührs H., Gudmundsson G.H. (2003). Expression of the cathelicidin LL-37 is modulated by short chain fatty acids in colonocytes: Relevance of signalling pathways. Gut.

[131] Schwab M., Reynders V., Shastri Y., Loitsch S., Stein J., Schröder O. (2007). Role of nuclear hormone receptors in butyrate-mediated up-regulation of the antimicrobial peptide cathelicidin in epithelial colorectal cells. Mol. Immunol..

[132] Steinmann J., Halldórsson S., Agerberth B., Gudmundsson G.H. (2009). Phenylbutyrate Induces Antimicrobial Peptide Expression. Antimicrob. Agents Chemother..

[133] Peric M., Koglin S., Dombrowski Y., Groß K., Bradac E., Ruzicka T., Schauber J. (2009). VDR and MEK-ERK dependent induction of the antimicrobial peptide cathelicidin in keratinocytes by lithocholic acid. Mol. Immunol..

[134] Baker R.G., Hayden M.S., Ghosh S. (2011). NF-κB, inflammation, and metabolic disease. Cell Metab..

[135] Fehlbaum P., Rao M., Zasloff M., Anderson G.M. (2000). An essential amino acid induces epithelial β-defensin expression. Proc. Natl. Acad. Sci. USA.

[136] Shao Z.-J., Zheng X.-W., Feng T., Huang J., Chen J., Wu Y.-Y., Zhou L.-M., Tu W.-W., Li H. (2012). Andrographolide exerted its antimicrobial effects by upregulation of human β-defensin-2 induced through p38 MAPK and NF-κB pathway in human lung epithelial cells. Can. J. Physiol. Pharmacol..

[137] Gan Y., Cui X., Ma T., Liu Y., Li A., Huang M. (2014). Paeoniflorin Upregulates β-Defensin-2 Expression in Human Bronchial Epithelial Cell Through the p38 MAPK, ERK, and NF-κB Signaling Pathways. Inflammation.

[138] Xiong W.-B., Shao Z.-J., Xiong Y., Chen J., Sun Y., Zhu L., Zhou L.-M. (2015). Dehydroandrographolide enhances innate immunity of intestinal tract through up-regulation the expression of hBD-2. DARU J. Pharm. Sci..

[139] Park K., Elias P.M., Hupe M., Borkowski A.W., Gallo R.L., Shin K.-O., Lee Y.-M., Holleran W.M., Uchida Y. (2013). Resveratrol Stimulates Sphingosine-1-Phosphate Signaling of Cathelicidin Production. J. Investig. Dermatol..

[140] Park K., Kim Y.-I., Shin K.-O., Seo H.S., Kim J.Y., Mann T., Oda Y., Lee Y.-M., Holleran W.M., Elias P.M. (2014). The dietary ingredient, genistein, stimulates cathelicidin antimicrobial peptide expression through a novel S1P-dependent mechanism. J. Nutr. Biochem..

[141] Xiong H., Guo B., Gan Z., Song D., Lu Z., Yi H., Wu Y., Wang Y., Du H. (2016). Butyrate upregulates endogenous host defense peptides to enhance disease resistance in piglets via histone deacetylase inhibition. Sci. Rep..

[142] Wang J., Huang N., Xiong J., Wei H., Jiang S., Peng J. (2018). Caprylic acid and nonanoic acid upregulate endogenous host defense peptides to enhance intestinal epithelial immunological barrier function via histone deacetylase inhibition. Int. Immunopharmacol..

[143] Andou A., Takehana K. (2009). Regulatory Roles of Amino Acids in Immune Response. Curr. Rheumatol. Rev..

[144] Sun J., Furio L., Mecheri R., van der Does A.M., Lundeberg E., Saveanu L., Chen Y., van Endert P., Agerberth B., Diana J. (2015). Pancreatic β-Cells Limit Autoimmune Diabetes via an Immunoregulatory Antimicrobial Peptide Expressed under the Influence of the Gut Microbiota. Immunity.

[145] Sherman H., Chapnik N., Froy O. (2006). Albumin and amino acids upregulate the expression of human beta-defensin 1. Mol. Immunol..

[146] Mao X., Qi S., Yu B., Huang Z., Chen H., Mao Q., Han G., Chen D. (2012). Dietary l-arginine supplementation enhances porcine β-defensins gene expression in some tissues of weaned pigs. Livest. Sci..

[147] Melano I., Kuo L.-L., Lo Y.-C., Sung P.-W., Tien N., Su W.-C. (2021). Effects of Basic Amino Acids and Their Derivatives on SARS-CoV-2 and Influenza-A Virus Infection. Viruses.

[148] Sunkara L.T., Jiang W., Zhang G. (2012). Modulation of Antimicrobial Host Defense Peptide Gene Expression by Free Fatty Acids. PLoS ONE.

[149] Alva-Murillo N., Ochoa-Zarzosa A., López-Meza J.E. (2012). Short chain fatty acids (propionic and hexanoic) decrease Staphylococcus aureus internalization into bovine mammary epithelial cells and modulate antimicrobial peptide expression. Veter- Microbiol..

[150] Roy C., Mandal S.M., Mondal S.K., Mukherjee S., Mapder T., Ghosh W., Chakraborty R. (2020). Trends of mutation accumulation across global SARS-CoV-2 genomes: Implications for the evolution of the novel coronavirus. Genomics.

[151] Baindara P., Ghosh A.K., Mandal S.M. (2020). Coevolution of Resistance Against Antimicrobial Peptides. Microb. Drug Resist..

[152] Bentley-Hewitt K.L., Blatchford P.A., Parkar S.G., Ansell J., Pernthaner A. (2012). Digested and Fermented Green Kiwifruit Increases Human β-Defensin 1 and 2 Production In vitro. Plant Foods Hum. Nutr..

[153] Jiang W., Sunkara L.T., Zeng X., Deng Z., Myers S.M., Zhang G. (2013). Differential regulation of human cathelicidin LL-37 by free fatty acids and their analogs. Peptides.

[154] Nylén F., Miraglia E., Cederlund A., Ottosson H., Strömberg R., Gudmundsson G.H., Agerberth B. (2013). Boosting innate immunity: Development and validation of a cell-based screening assay to identify LL-37 inducers. J. Endotoxin Res..

[155] Cederlund A., Kai-Larsen Y., Printz G., Yoshio H., Alvelius G., Lagercrantz H., Strömberg R., Jörnvall H., Gudmundsson G.H., Agerberth B. (2013). Lactose in Human Breast Milk an Inducer of Innate Immunity with Implications for a Role in Intestinal Homeostasis. PLoS ONE.

[156] Malik A.N., Al-Kafaji G. (2007). Glucose regulation of β-defensin-1 mRNA in human renal cells. Biochem. Biophys. Res. Commun..

[157] Díaz L.A.C., Miramontes M.G.F., Hurtado P.C., Allen K., Ávila M.G., de Oca E.P.M. (2015). Ascorbic Acid, Ultraviolet C Rays, and Glucose but not Hyperthermia Are Elicitors of Humanβ-Defensin 1 mRNA in Normal Keratinocytes. BioMed Res. Int..

[158] Barnea M., Madar Z., Froy O. (2008). Glucose and insulin are needed for optimal defensin expression in human cell lines. Biochem. Biophys. Res. Commun..

[159] Lan C.-C.E., Wu C.-S., Huang S.-M., Kuo H.-Y., Wu I.-H., Wen C.-H., Chai C.-Y., Fang A.-H., Chen G.-S. (2011). High-Glucose Environment Inhibits p38MAPK Signaling and Reduces Human β-3 Expression in Keratinocytes. Mol. Med..

[160] Donnarumma G., Buommino E., Baroni A., Auricchio L., De Filippis A., Cozza V., Msika P., Piccardi N., Tufano M.A. (2007). Effects of AV119, a natural sugar from avocado, on Malassezia furfur invasiveness and on the expression of HBD-2 and cytokines in human keratinocytes. Exp. Dermatol..

[161] Paoletti I., Buommino E., Fusco A., Baudouin C., Msika P., Tufano M.A., Baroni A., Donnarumma G. (2012). Patented natural avocado sugar modulates the HBD-2 and HBD-3 expression in human keratinocytes through Toll-like receptor-2 and ERK/MAPK activation. Arch. Dermatol. Res..

[162] Žugčić T., Abdelkebir R., Alcantara C., Collado M.C., Garcia-Perez J.V., Meléndez-Martínez A.J., Režek Jambrak A., Lorenzo J.M., Barba F.J. (2019). From extraction of valuable compounds to health promoting benefits of olive leaves through bioaccessibility, bioavailability and impact on gut microbiota. Trends Food Sci. Technol..

[163] Van Hai N. (2015). The use of medicinal plants as immunostimulants in aquaculture: A review. Aquaculture.

[164] Cabrera C., Artacho R., Giménez R. (2006). Beneficial Effects of Green Tea—A Review. J. Am. Coll. Nutr..

[165] Cooper R. (2011). Green tea and theanine: Health benefits. Int. J. Food Sci. Nutr..

[166] Bedran T.B.L., Feghali K., Zhao L., Spolidorio D.M.P., Grenier D. (2013). Green tea extract and its major constituent, epigallocatechin-3-gallate, induce epithelial beta-defensin secretion and prevent beta-defensin degradation by *Porphyromonas gingivalis*. J. Periodontal Res..

[167] Bedran T.B.L., Morin M.-P., Spolidorio D.P., Grenier D. (2015). Black Tea Extract and Its Theaflavin Derivatives Inhibit the Growth of Periodontopathogens and Modulate Interleukin-8 and β-Defensin Secretion in Oral Epithelial Cells. PLoS ONE.

[168] Promsong A., Chung W.O., Satthakarn S., Nittayananta W. (2014). Ellagic acid modulates the expression of oral innate immune mediators: Potential role in mucosal protection. J. Oral Pathol. Med..

[169] Lin C.-W., Tsai F.-J., Tsai C.-H., Lai C.-C., Wan L., Ho T.-Y., Hsieh C.-C., Chao P.-D.L. (2005). Anti-SARS coronavirus 3C-like protease effects of Isatis indigotica root and plant-derived phenolic compounds. Antivir. Res..

[170] Mao X., Qi S., Yu B., He J., Yu J., Chen D. (2012). Zn2+ and l-isoleucine induce the expressions of porcine β-defensins in IPEC-J2 cells. Mol. Biol. Rep..

[171] Pernet I., Reymermier C., Guezennec A., Branka J.-E., Guesnet J., Perrier E., Dezutter-Dambuyant C., Schmitt D., Viac J. (2003). Calcium triggers beta-defensin (hBD-2 and hBD-3) and chemokine macrophage inflammatory protein-3alpha (MIP-3alpha/CCL20) expression in monolayers of activated human keratinocytes. Exp. Dermatol..

[172] Chen K., Chen H., Faas M.M., de Haan B.J., Li J., Xiao P., Zhang H., Diana J., de Vos P., Sun J. (2017). Specific inulin-type fructan fibers protect against autoimmune diabetes by modulating gut immunity, barrier function, and microbiota homeostasis. Mol. Nutr. Food Res..

[173] He Y., Wu C., Li J., Li H., Sun Z., Zhang H., De Vos P., Pan L.-L., Sun J. (2017). Inulin-Type Fructans Modulates Pancreatic–Gut Innate Immune Responses and Gut Barrier Integrity during Experimental Acute Pancreatitis in a Chain Length-Dependent Manner. Front. Immunol..

[174] Termén S., Tollin M., Rodriguez E., Sveinsdóttir S.H., Jóhannesson B., Cederlund A., Sjövall J., Agerberth B., Gudmundsson G.H. (2008). PU.1 and bacterial metabolites regulate the human gene CAMP encoding antimicrobial peptide LL-37 in colon epithelial cells. Mol. Immunol..

[175] Ottosson H., Nylén F., Sarker P., Miraglia E., Bergman P., Gudmundsson G.H., Raqib R., Agerberth B., Strömberg R. (2016). Potent Inducers of Endogenous Antimicrobial Peptides for Host Directed Therapy of Infections. Sci. Rep..

[176] Holstein J., Fehrenbacher B., Brück J., Müller-Hermelink E., Schäfer I., Carevic M., Schittek B., Schaller M., Ghoreschi K., Eberle F.C. (2017). Anthralin modulates the expression pattern of cytokeratins and antimicrobial peptides by psoriatic keratinocytes. J. Dermatol. Sci..

[177] Lyu W., Deng Z., Sunkara L.T., Becker S., Robinson K., Matts R., Zhang G. (2018). High Throughput Screening for Natural Host Defense Peptide-Inducing Compounds as Novel Alternatives to Antibiotics. Front. Cell. Infect. Microbiol..

[178] Zhao Y., Chen F., Wu W., Sun M., Bilotta A.J., Yao S., Xiao Y., Huang X., Eaves-Pyles T.D., Golovko G. (2018). GPR43 mediates microbiota metabolite SCFA regulation of antimicrobial peptide expression in intestinal epithelial cells via activation of mTOR and STAT3. Mucosal Immunol..

[179] Guo C., Rosoha E., Lowry M.B., Borregaard N., Gombart A.F. (2012). Curcumin induces human cathelicidin antimicrobial peptide gene expression through a vitamin D receptor-independent pathway. J. Nutr. Biochem..

[180] Kauko O., Laajala T.D., Jumppanen M., Hintsanen P., Suni V., Haapaniemi P., Corthals G., Aittokallio T., Westermarck J., Imanishi S. (2015). Label-free quantitative phosphoproteomics with novel pairwise abundance normalization reveals synergistic RAS and CIP2A signaling. Sci. Rep..

[181] Bader G.D., Hogue C.W.V. (2003). An automated method for finding molecular complexes in large protein interaction networks. BMC Bioinform..

[182] Bindea G., Mlecnik B., Hackl H., Charoentong P., Tosolini M., Kirilovsky A., Fridman W.-H., Pagès F., Trajanoski Z., Galon J. (2009). ClueGO: A Cytoscape plug-in to decipher functionally grouped gene ontology and pathway annotation networks. Bioinformatics.

[183] Janky R., Verfaillie A., Imrichova H., Van de Sande B., Standaert L., Christiaens V., Hulselmans G., Herten K., Sanchez M.N., Potier D. (2014). iRegulon: From a Gene List to a Gene Regulatory Network Using Large Motif and Track Collections. PLOS Comput. Biol..

[184] Baindara P., Agrawal S., Mandal S.M. (2020). Host-directed therapies: A potential solution to combat COVID-19. Expert Opin. Biol. Ther..

[185] Mandal S.M., Panda S. (2020). Inhaler with electrostatic sterilizer and use of cationic amphiphilic peptides may accelerate recovery from COVID-19. Biotechniques.

[186] Mandal S. (2020). Peptide targets to SARS-CoV-2. J. Glob. Infect. Dis..

[187] Chowdhury T., Baindara P., Mandal S.M. (2020). LPD-12: A promising lipopeptide to control COVID-19. Int. J. Antimicrob. Agents.

