# Supplementary material for: Preventing Respiratory Viral Diseases with Antimicrobial Peptide Master Regulators in the Lung Airway Habitat
Source: Clin Pract. 2023 Jan 16;13(1):125–47. doi: 10.3390/clinpract13010012 (PMC9844411; doi:10.3390/clinpract13010012)

Review

Preventing Respiratory Viral Diseases with Antimicrobial Peptide Master Regulators in the Lung Airway Habitat

Piyush Baindara [1], Sriradha Ganguli [2], Ranadhir Chakraborty [2] and Santi M. Mandal [3,*]

[1] Department of Radiation Oncology, University of Missouri, Columbia 65211, Missouri, United States
[2] Department of Biotechnology, OMICS Laboratory, University of North Bengal, P.O. NBU, Siliguri 734013, West Bengal, India
[3] Department of Biotechnology, Indian Institute of Technology Kharagpur, Kharagpur 721302, India

* Correspondence: mandalsm@gmail.com

Figure S1. Functional annotation of genes of module 1 using Cytoscape Plugin ClueGO. Yellow connecting lines show viral interactions with cytokines and cytokine receptors while various components of the immune response during viral infections are shown with purple, blue, green, sea green, and grey circles.

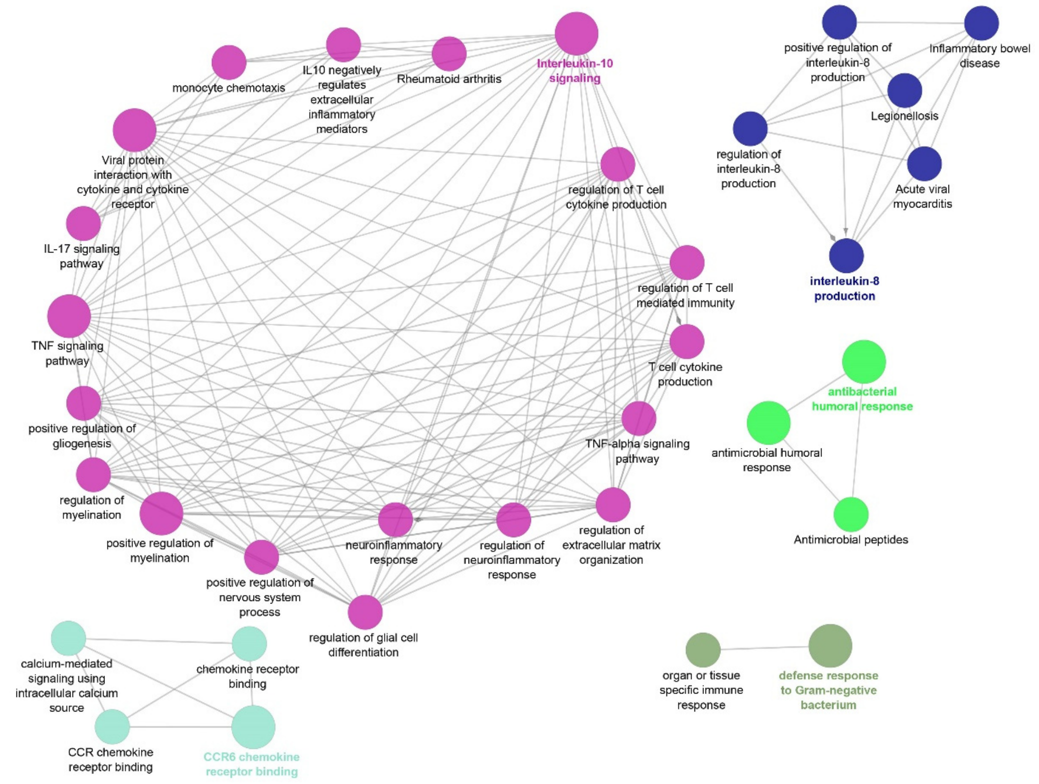

Figure S2. Functional annotation of genes of module 2 using Cytoscape Plugin ClueGO. Yellow connecting lines show viral interactions with cytokines and cytokine receptors while various components of the immune response during viral infections are shown with red, purple, blue, green, sea green, and grey circles.

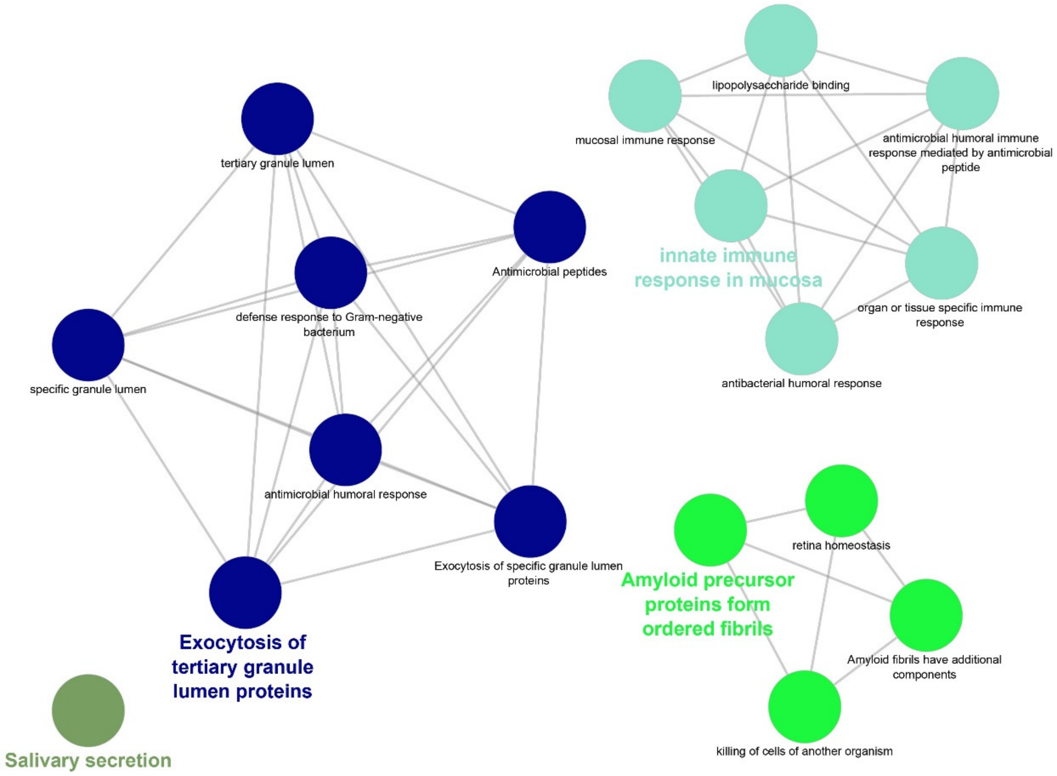

Figure S3. Regulatory and metatargetome analysis of module 1. (A) Regulatory analysis of module 1 using Cytoscape plugin iRegulon. The table shows the details of transcription factors involved in the regulation of module1 (B) Metatargetome analysis of TFs targeting genes of module1.

(A)

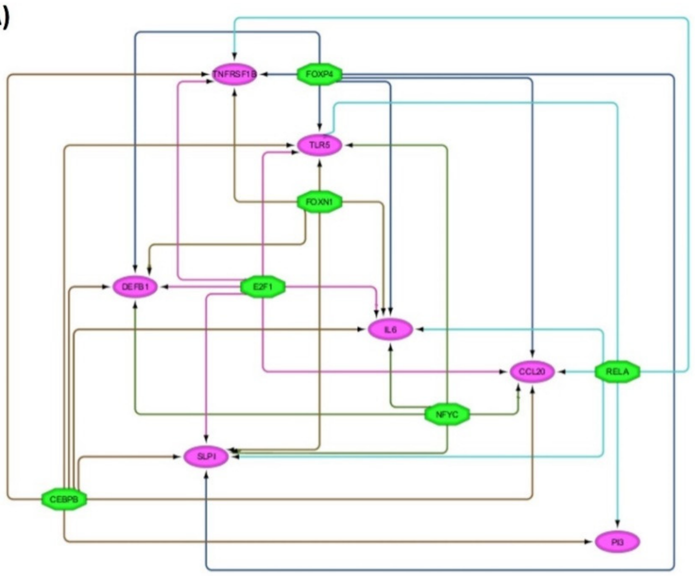

TF	NES	#Targets	#Motifs/Tracks
CEBPD	8.105	7	7
RELA	9.358	6	11
CEBPE(E2F1)	6.290	6	11
FOXP4	4.720	6	11
CEBPA(NFYC)	8.705	5	7
FOXN1	7.661	5	7

(B)

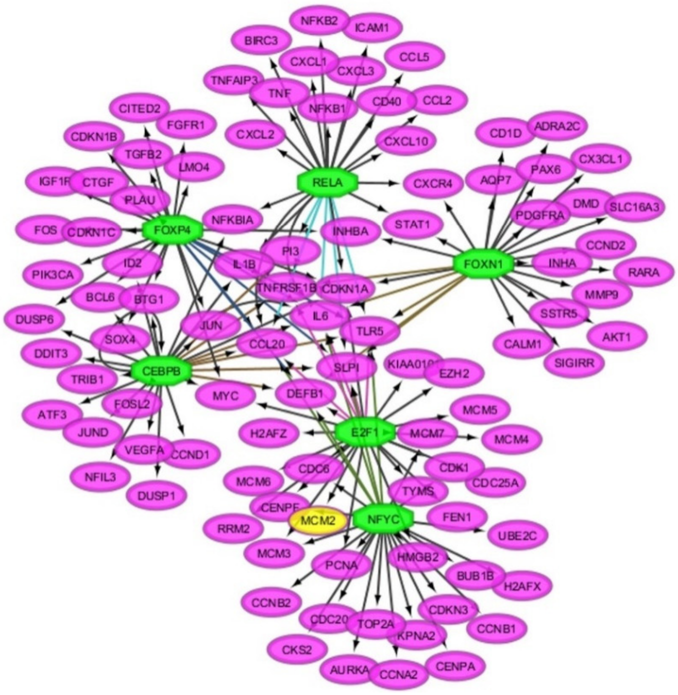

Figure S4. Regulatory and metatargetome analysis of module 2. (A) Regulatory analysis of module 2 using Cytoscape plugin iRegulon. The table shows the details of transcription factors involved in the regulation of module 2 (B) Metatargetome analysis of TFs targeting genes of module 2.

(A)

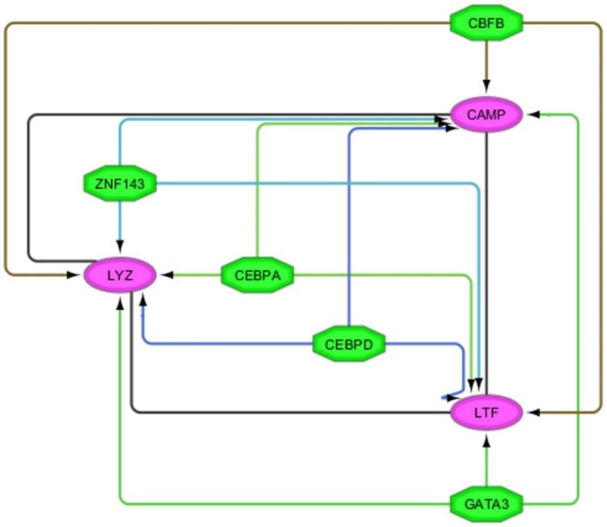

TF	NES	#Targets	#Motifs/Tracks
CBFB	8.490	3	10
CEBPD	8.201	3	7
GATA3	7.583	3	4
CEBPA	6.834	3	2
ZNF143	6.334	3	2

(B)

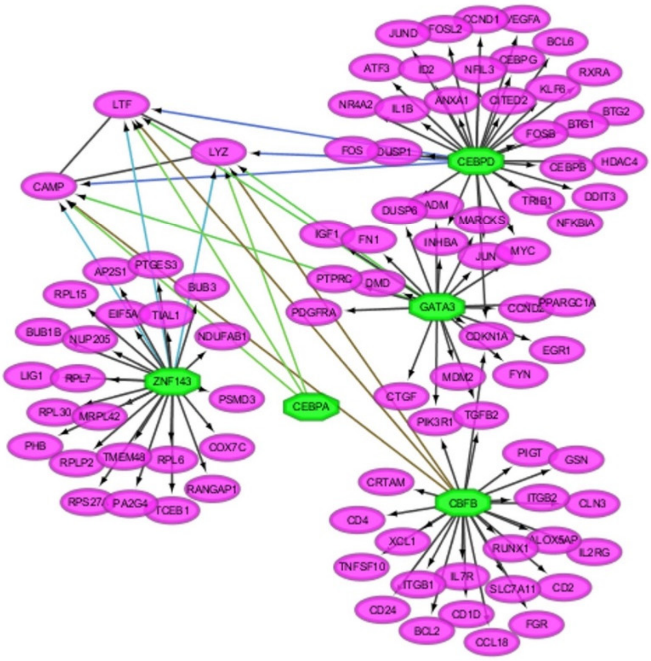

Supplement: Supplementary file 1 [file clinpract-13-00012-s001.zip › clinpract-2056715-supplementary.pdf]
